# Ecogenomic Perspectives on Domains of Unknown Function: Correlation-Based Exploration of Marine Metagenomes

**DOI:** 10.1371/journal.pone.0050869

**Published:** 2013-03-14

**Authors:** Pier Luigi Buttigieg, Wolfgang Hankeln, Ivaylo Kostadinov, Renzo Kottmann, Pelin Yilmaz, Melissa Beth Duhaime, Frank Oliver Glöckner

**Affiliations:** 1 Microbial Genomics and Bioinformatics Group, Max Planck Institute for Marine Microbiology, Bremen, Germany; 2 Jacobs University gGmbH, Bremen, Germany; 3 Departament de Biologia Marina i Oceanografia, Institut de Ciències del Mar, Barcelona, Spain; 4 Department of Ecology and Evolutionary Biology, University of Michigan, Ann Arbor, Michigan, United States of America; Miami University, United States of America

## Abstract

**Background:**

The proportion of conserved DNA sequences with no clear function is steadily growing in bioinformatics databases. Studies of sequence and structural homology have indicated that many uncharacterized protein domain sequences are variants of functionally described domains. If these variants promote an organism's ecological fitness, they are likely to be conserved in the genome of its progeny and the population at large. The genetic composition of microbial communities in their native ecosystems is accessible through metagenomics. We hypothesize the co-variation of protein domain sequences across metagenomes from similar ecosystems will provide insights into their potential roles and aid further investigation.

**Methodology/Principal findings:**

We calculated the correlation of Pfam protein domain sequences across the Global Ocean Sampling metagenome collection, employing conservative detection and correlation thresholds to limit results to well-supported hits and associations. We then examined intercorrelations between domains of unknown function (DUFs) and domains involved in known metabolic pathways using network visualization and cluster-detection tools. We used a cautious “guilty-by-association” approach, referencing knowledge-level resources to identify and discuss associations that offer insight into DUF function. We observed numerous DUFs associated to photobiologically active domains and prevalent in the *Cyanobacteria*. Other clusters included DUFs associated with DNA maintenance and repair, inorganic nutrient metabolism, and sodium-translocating transport domains. We also observed a number of clusters reflecting known metabolic associations and cases that predicted functional reclassification of DUFs.

**Conclusion/Significance:**

Critically examining domain covariation across metagenomic datasets can grant new perspectives on the roles and associations of DUFs in an ecological setting. Targeted attempts at DUF characterization in the laboratory or *in silico* may draw from these insights and opportunities to discover new associations and corroborate existing ones will arise as more large-scale metagenomic datasets emerge.

## Introduction

In recent years, genomic sequencing projects have revealed a large number of novel genes across a wide range of organisms and environments. Many of these have poor sequence-level similarity to genes that have been characterized in a laboratory setting and, consequently, have not been annotated with functional roles. These ‘hypothetical’ genes are becoming increasingly prevalent in bioinformatics databases. For example, the Pfam 24 database [1] stored some 11,912 protein domain families derived from conserved sequence data with ∼26% dubbed “domains of unknown function” (DUFs). This proportion is predicted to soon overtake that of functionally characterized domains [2], and calls for community action [3] and cross-disciplinary efforts [4] towards their identification have been made.

Several groups have employed biochemical and molecular techniques in DUF characterization. Among these, Deng et al. [5] characterized members of a Pfam family, formerly known as DUF62, as S-adenosyl-l-methionine hydroxide adenosyltransferases using a range of enzymatic analyses. Similarly, Weinitschke et al. characterized members of the DUF81 family as the sulfite exporter TauE by examining co-transcription of genes in the metabolism of C_2_ sulfonates [6]. Computational approaches have also been used to aid DUF characterization. Goonesekere et al. [7] applied secondary structure analysis and the use of three-dimensional homology models to functionally annotate 8 DUFs. On a larger scale, Jaroszewski et al. [8] used structural genomics approaches to determine the three-dimensional structures for more than 250 DUF families, granting insights into their potential activities. This latter study reported that the majority of DUFs analyzed were either divergent structural variants of well-characterized families or showed notable substructure similarity to known proteins. The authors inferred that these variations may have been conserved as they extended an organism's functional repertoire in an ecologically adaptive manner. Here, we explored a marine metagenomic dataset to gain an ecological perspective on DUF functionality.

Metagenomics allows insight into an entire ecological community's genetic content [9], [10], providing a rich source of *in situ* sequence data from a range of ecosystems. These metagenomic resources are ripe for mining, with intra-ecosystem variation in microbial function cited as a high-value target for exploration [11]. If genomic conservation of DUFs is adaptive in nature, then it is probable that these DUFs are co-selected with other domains linked to that adaptation. We thus hypothesized that the abundances of co-selected domains will co-vary across metagenomes from similar ecosystems. By identifying co-varying groups of domains, it may be possible to speculate on DUF functionality using a ‘guilty-by-association’ approach [12]. This approach is often used in the identification of novel metabolic modules by relating experimental perturbations to subsequent gene expression patterns [13]. In this model, genes with similar responses are grouped into putative modules to guide more rigorous future investigation. Further, correlative approaches have been employed to explore ecosystem-level interactions between microbial taxa and their environment [14], [15].

Here, we propose that correlations of protein domain sequences found in intra-ecosystem metagenomic data may provide insight into the functional roles of DUFs. We critically examined the correlation of protein domains of known and unknown function across the Global Ocean Sampling (GOS) collection [16], comprising a globally distributed set of epipelagic microbial community metagenomes. We found several sets of associations that show promise in guiding DUF characterization in an experimental setting.

## Results and Discussion

### Pfam versions in analysis and interpretation

Here, we queried raw sequences of selected GOS metagenomes against version 24 of the Pfam database [1]. Of the 3,069 DUFs in Pfam v24, we detected 2,531 in our analysis. In subsequent Pfam database releases, many models and their functional descriptions have been updated to reflect the growing knowledge surrounding them. The associations presented here are best validated by domain-level knowledge. We thus referred to Pfam v26 [17] when discussing our results, allowing several correlative hypotheses to be evaluated against later functional characterizations.

### Correlation analysis

Removal of rows (sites) and columns (Pfams) dominated by zero abundances was performed to decrease the sparseness of the dataset and focus on observed variation. The dataset was thus reduced from a matrix of 80 GOS metagenomes and 3,587 Pfam domains to 69 sites and 1,863 Pfam domains. A total of 670 of the domains retained were DUFs. We standardized abundances by their site maxima to mitigate the effect of differing sample size across metagenomes; however, the co-variation of domain abundances due to sample size may still provide insights into their potential associations, assuming proportionality. Thus, we used the unstandardized matrix (UM) and its standardized counterpart (SM) in further analysis. Totals of 975 (UM) and 167 (SM) Pfams were retained following removal of those with no correlations stronger than a rho of 0.80 and Bonferroni-corrected P-values below 1×10^−6^. These included 225 (UM) and 75 (SM) DUFs. Correlation of domains in the UM resulted in largely Gaussian distribution of rho coefficients (mean≈0.41, s.d.≈0.26); however, a slight second peak around a rho of 0.80 was observed (**Figure S1**). Correlation of domains in the SM displayed a more Gaussian distribution of rho values (mean≈0.03, s.d.≈0.19); however, with a positive skew (**Figure S2**). Based on these distributions, we chose a correlation threshold and conservative significance values to focus on robust associations.

In the UM, a total of 94 DUFs showed biased correlations to Pfam domains assigned to a single metabolic category (Table 1). Of these, 56 were biased towards photobiological processes (**Tables S1 and S2**), many of which were also associated with a septum formation inhibitor domain (MinC_C) involved in the control of cell division. In the analysis of the SM, 48 DUFs showed biased correlations, with 45 in favor of photobiological processes (**Table S3**). Of these 45, 15 showed some bias (≥40% of the maximum correlated category) towards domains in carbohydrate and/or coenzyme metabolism. The three remaining DUFs were associated to domains in inorganic ion transport and metabolism (DUF1008); carbohydrate transport and metabolism (DUF111); and transcription (DUF37). Such biased connectivity of DUFs to domains in a particular functional category is not a guarantee of related function; however, it provides associative context for hypothesis generation.

**Table 1:** DUFs* exhibiting bias in correlative associations to metabolic categories (unstandardized data).

DUF	Primary category	Secondary category (fraction of primary category)
DUF59	TransR	AA (0.44)
DUF3429		AA (0.44)
DUF805		AA (0.48)
DUF140		CoE (0.45)
DUF354		CoE (0.5)
DUF151		RRR (0.43)
DUF192		RRR (0.44)
DUF87		RRR (0.46)
DUF37		Transcr (0.50)
DUF1730		None
DUF2805		None
DUF3159	AA	Carb, E, Lip, Nuc (0.50)
DUF521		CoE, E, TransR (0.50)
DUF1329		None
DUF208		None
DUF2141		None
DUF2899		None
DUF403		None
DUF407		None
DUF490	CoE	CWME, E, Photo, Sec (0.50)
DUF1499		None
DUF1820		None
DUF92		None
DUF2062	E	AA, Carb, CoE, RRR (0.50)
DUF137		None
DUF74		None
DUF2130	Carb	CoE, Photo (0.50)
DUF897		None
DUF1445	Lip	None
DUF501		None
DUF1732	PostModChaps	Carb, Nuc (0.33)
DUF1385		None
DUF1008	Ion	None
DUF88	Nuc	None
DUF2334	RRR	None
DUF429	Sec	None
DUF1907	Sig	None
DUF2779	Transcr	CWME, PostModChaps (0.50)

* DUFs with primary connectivity to photobiology domains (n = 56) present in **Table S2**.

AA: Amino acid transport and metabolism.

Carb: Carbohydrate transport and metabolism.

CellDiv: Cell cycle control, cell division, chromosome partitioning.

CoE: Coenzyme transport and metabolism.

CWME: Cell wall, membrane, and envelope biogenesis.

Def: Defence mechanisms.

E: Energy production and conversion.

Ion: Inorganic ion transport and metabolism.

Lip: Lipid transport and metabolism.

Nuc: Nucleotide transport and metabolism.

Photo: Photobiology.

PostModChaps: Posttranslational modification, protein turnover, chaperones.

RRR: Replication, recombination, and repair.

Sec: Secondary metabolite biosynthesis, transport, and catabolism.

Sig: Signal transduction mechanisms.

Transcr: Transcription.

TransR: Translation, ribosomal structure and biogenesis.

### Network exploration

#### General network characteristics

**Figure 1:**
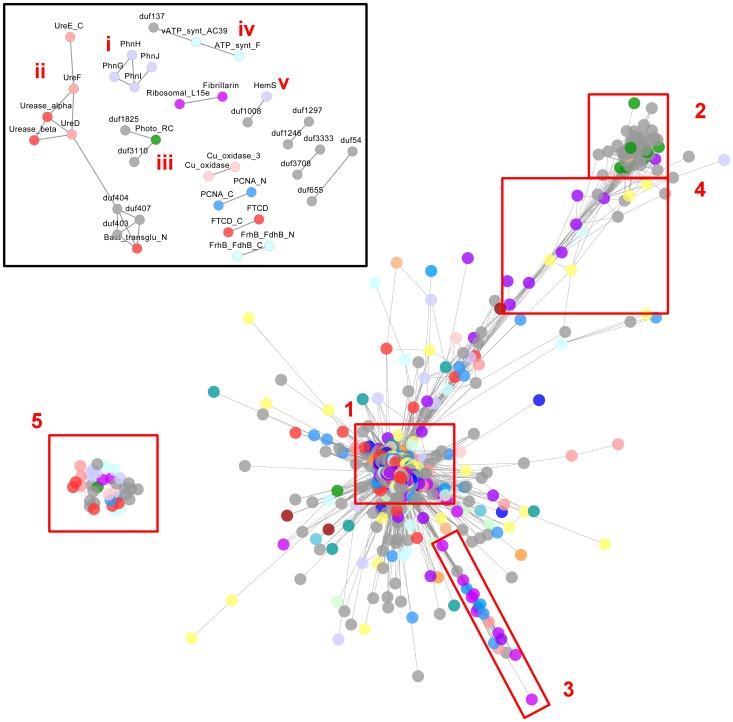
Force-directed, spring-embedded network visualization of pairwise correlations between Pfam domain abundances across selected GOS metagenomes. Nodes represent Pfam domains and edges correlations greater than a Spearman's rho of 0.80. Shorter edges indicate stronger correlations. A large network with two enmeshed regions (Box 1 and 2) bridged by a small number of nodes (Box 4) dominates the graph. Several small networks of functionally related nodes are also present (Box 5, inset). Node colors represent functional categories; refer to **Figure S7** for description. See text for detailed descriptions.

The networks generated from the correlation of the UM and SM described above comprised 975 nodes and 166,232 edges and 167 nodes and 1,897 edges, respectively. The network derived from the unstandardized abundances contained two regions of highly interconnected vertices, each with a mesh-like topology (**Figure 1**, Boxes 1 and 2; see **Figure S3** for node labels). The larger of these regions (Box 1) contained a core of highly interconnected nodes with domains from a wide variety of metabolic categories, as well as ‘spokes’ of less interconnected nodes. The highly-enmeshed topology of this large connected complex (LCC) prevents speculation on DUF function by visual inspection alone. However, a spoke of the UM LCC (**Figure 1**, Box 3) containing DUF2805 and DUF37 was defined by domains linked to protein biosynthesis at the level of translation (**Table 2**).

**Table 2:** Pfam domains contained in a prominent spoke of the UM-derived association network (Figure 1, Box 3).

Category	Pfam ID	Pfam comment (abridged)
CWME	LpxD	UDP-3-O-[3-hydroxymyristoyl] glucosamine N-acyltransferase catalyses an early step in lipid A biosynthesis. Members of this family also contain a hexapeptide repeat (Pfam:PF00132). This family constitutes the non-repeating region of LPXD proteins.
CoE	Porphobil_deamC	–
NA	DUF2805	This is a bacterial family of proteins with unknown function.
	DUF37	This domain is found in short (70 amino acid) hypothetical proteins from various bacteria. The domain contains three conserved cysteine residues. Swiss:Q44066 from Aeromonas hydrophila has been found to have hemolytic activity (unpublished).
PostModChaps	NifU	This is an alignment of the carboxy-terminal domain. This is the only common region between the NifU protein from nitrogen-fixing bacteria and rhodobacterial species. The biochemical function of NifU is unknown.
	SmpB	–
	Bac_DnaA_C	–
	DNA_gyraseB_C	The amino terminus of eukaryotic and prokaryotic DNA topoisomerase II are similar, but they have a different carboxyl terminus. The amino-terminal portion of the DNA gyrase B protein is thought to catalyse the ATP-dependent super-coiling of DNA. See Pfam:PF00204. The carboxyl-terminal end supports the complexation with the DNA gyrase A protein and the ATP-independent relaxation. This family also contains Topoisomerase IV. This is a bacterial enzyme that is closely related to DNA gyrase.
Transcr	RNA_pol_Rpb2_3	RNA polymerases catalyse the DNA dependent polymerisation of RNA. Prokaryotes contain a single RNA polymerase compared to three in eukaryotes (not including mitochondrial. and chloroplast polymerases). Domain 3, s also known as the fork domain and is proximal to catalytic site.
	Sigma70_r2	Region 2 of sigma-70 is the most conserved region of the entire protein. All members of this class of sigma-factor contain region 2. The high conservation is due to region 2 containing both the −10 promoter recognition helix and the primary core RNA polymerase binding determinant. The core binding helix, interacts with the clamp domain of the largest polymerase subunit, beta prime. The aromatic residues of the recognition helix, found at the C-terminus of this domain are though to mediate strand separation, thereby allowing transcription initiation.
TransR	B5	This domain is found in phenylalanine-tRNA synthetase beta subunits.
	Glu-tRNAGln	This is a family of Glu-tRNAGln amidotransferase C subunits. The Glu-tRNA Gln amidotransferase enzyme itself is an important translational fidelity mechanism replacing incorrectly charged Glu-tRNAGln with the correct Gln-tRANGln via transmidation of the misacylated Glu-tRNAGln. This activity supplements the lack of glutaminyl-tRNA synthetase activity in gram-positive eubacterteria, cyanobacteria, Archaea, and organelles.
	Phe_tRNA-synt_N	–
	Ribosomal_L11	–
	Ribosomal_L12	–
	Ribosomal_S17	–
	Ribosomal_S5	–
	RNase_PH_C	This family includes 3′-5′ exoribonucleases. Ribonuclease PH contains a single copy of this domain, and removes nucleotide residues following the -CCA terminus of tRNA. Polyribonucleotide nucleotidyltransferase (PNPase) contains two tandem copies of the domain. PNPase is involved in mRNA degradation in a 3′-5′ direction. The exosome is a 3′-5′ exoribonuclease complex that is required for 3′ processing of the 5.8S rRNA. Three of its five protein components, Swiss:P46948 Swiss:Q12277 and Swiss:P25359 contain a copy of this domain. Swiss:Q10205, a hypothetical protein from S. pombe appears to belong to an uncharacterised subfamily. This subfamily is found in both eukaryotes and archaebacteria.

Refer to Table 1, footnote for list of abbreviations.

#### Network regions with photobiological relevance

The smaller region of this network (**Figure 1**, Box 2) was dominated by DUFs and included ten domains implicated in photobiological processes (**Table S4**). These included a central photosystem II domain, a subunit of the photosystem I reaction center, a domain that targets precursors of light-harvesting prosthetic groups, and a proton-pumping domain essential for photoheterotrophic cell growth. The taxonomic distribution of the DUFs in this region (after Pfam v26) was heavily weighted toward the Cyanobacteria and other photobiologically active taxa, such as the Viridiplantae. It is premature to connect the DUFs in this region directly to phototrophic mechanisms; however, a photoresponsive mode of life may have some bearing on their function and association in marine metagenomes.

Additionally, we noted the domains bridging this putative module to the bulk of the LCC were also photobiologically relevant (**Figure 1**, Box 4; **Table S5**). For example, eight domains were involved in cobalamin metabolism and the large and small subunits of RuBisCO were also present. Cobalamin (vitamin B_12_) is characterized by a corrin ring, which is chemically similar to the porphyrin ring found in heme, chlorophyll, and cytochrome. Further, cobalamin-dependent methyltransferases are involved in C1 metabolism and CO_2_ fixation [18].

**Figure 2:**
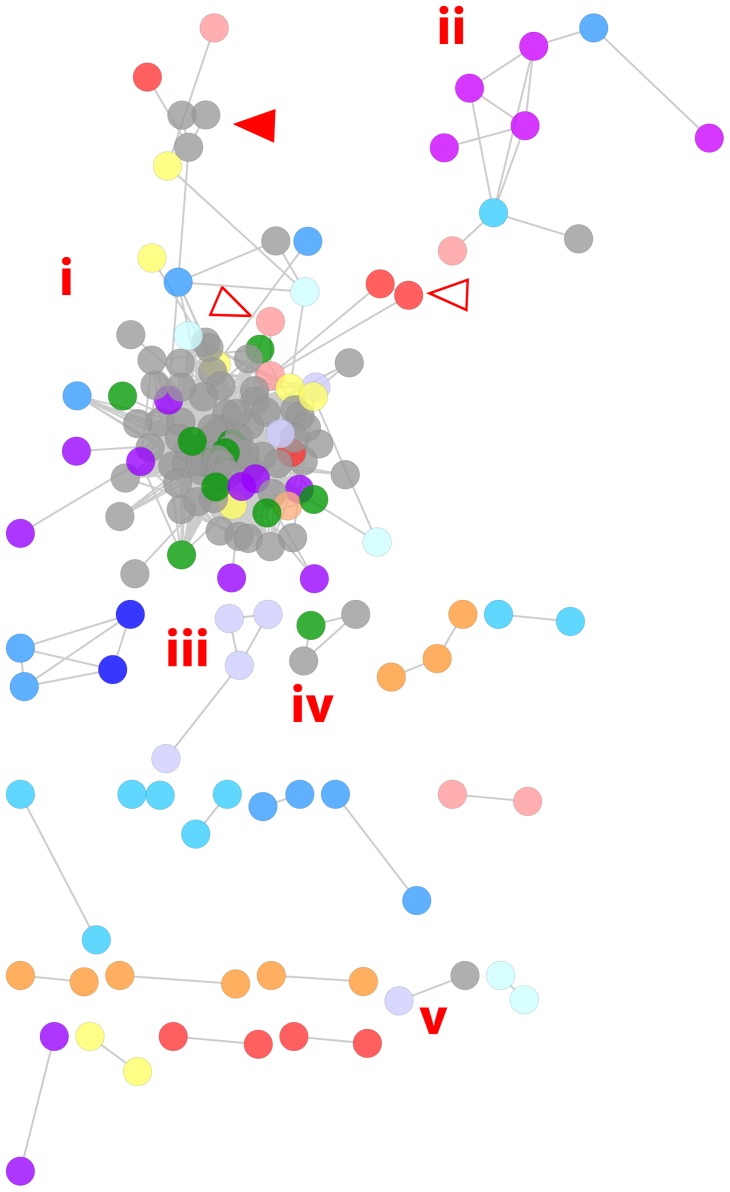
Force-directed, spring-embedded network visualization of pairwise correlations between standardized Pfam domain abundances across selected GOS metagenomes. Abundances were standardized by site maxima. Nodes represent Pfam domains and edges correlations greater than a Spearman's rho of 0.80. Shorter edges indicate stronger correlations. The largest network (i) is dominated by DUFs and domains linked to photobiological processes. Domains linked to urea metabolism were also present in this network (hollow arrowheads). Smaller networks featured domains linked to translation (ii), phosphonate metabolism (iii), and cyanophage activity (iv). Numerous pairs of functionally related domains were also present. Node colors represent functional categories; refer to **Figure S7** for description. See text for detailed descriptions.

We observed an SM-derived network (**Figure 2, i**; see **Figure S4** for node labels) with similar membership to the bridge and photobiological regions discussed above (**Table S6**). This network featured 112 Pfams, which included DUFs (n = 72), photoreactive domains (n = 10), and coenzyme transport and metabolism domains (n = 8). We also observed a network (**Figure 2**, ii) with similar membership to the spoke comprising domains related to protein biosynthesis discussed above (**Figure 1**, Box 4).

#### Networks with known functional relatedness

A number of comparatively small UM-derived networks were also observed (**Figure 1**, Box 5 & inset), including domain groups known to be functionally related. Detection of correlative associations between such domains is encouraging and lends merit to the potential of metagenomic datasets in functional module detection. For example, we observed a group of phosphonate metabolism domains as well as a group of urea metabolism domains in the UM-derived networks (**Figure 1**, inset, i and ii resp.). Analysis of the standardized data also revealed the phosphonate network (**Figure 2**, iii), as well as some of the urea metabolic domains noted above, embedded in the LCC (**Figure 2**, hollow arrowheads). Phosphonate and urea are significant components of the marine dissolved organic matter pool and numerous microbial genera, such as *Prochlorococcus*, possess corresponding uptake and utilization capacities [19]–[21]. The urea metabolism domains were associated with a bacterial transglutaminase-like domain (Bact_transglu_N), DUF403, DUF404, and DUF407 in the UM-derived networks. These latter domains occurred on the periphery of the SM-derived LCC (**Figure 2**, solid arrowhead). In Pfam 26, DUF407 has been reclassified as a circularly permuted ATP-grasp family (CP_ATPgrasp_1) and DUF404 has been merged with this family. Further, Pfam architectures place instances of DUF403, now referred to as Alpha-E, in proximity to both Bact_transglu_N and CP_ATPgrasp_1 in microbial genomes. Goonesekere et al. [7] also observed that DUFs 404 and 407 co-occur. Together, these domains function in a peptide synthesis/modification system [22].

We observed a number of associations between N- and C-terminal domain abundances in analysis of both the SM and UM. Examples include arginine-tRNA-protein transferase (ATE_N; ATE_C), coenzyme F420 hydrogenase (FrhB_FdhB_N; FrhB_FdhB_C), and domains involved in eukaryotic DNA polymerase processivity (PCNA_N; PCNA_C).

We also observed several small networks with DUFs associated to domains that may shed light on their roles. Notably, a haemin-degrading domain was associated with DUF1008 in analysis of both the SM (**Figure 2**, v) and UM (**Figure 1**, inset, v). DUF1008 has been reclassified as a haem utilization ChuX/HutX domain in Pfam v26, which corroborates this correlative association. Further, a photoreaction center domain was associated with DUF1825 and DUF3110 in both the SM (**Figure 2**, iv) and UM analyses (**Figure 1**, inset, iii). These DUFs principally occur in the *Cyanobacteria*; however, their specific correlation with the photoreaction center domain is likely caused by the co-occurrence of these domains in marine cyanophages, which infect the genera *Synechococcus* and *Prochlorococcus*. Previous work has identified a number of cyanophage-borne photosynthetic genes which are transferred to their hosts, including *psbA* and *psbD* [23], both of which bear the Photo_RC domain. Further, DUF3708, annotated as a phosphate ATP-binding cassette transporter, was associated with DUF3333 in the UM-derived networks. Both these DUFs are prevalent in the *Alphaproteobacteria* and Pfam architectures note them in proximity to the inner membrane component of a binding-protein-dependent transport system (BPD_transp_1). DUF137 was associated with two ATP synthase subunits. We observed these three domains to be prevalent in the *Archaea*, thus, attributing DUF137 with ATP synthase activity is premature, as taxonomic restriction may also explain their correlation. A similar explanation can be applied to the association of DUF655 and DUF54, the former domain reportedly resembling a ribosomal protein [24]. DUF1297 and DUF1246, associated as a pair, share several Pfam architectures, are listed as Pfam interaction partners, and are also prominent in the *Archaea*. Similarly, a pair comprising DUF126 and DUF521 shared similar taxonomic distributions, occurring primarily in the *Bacteria* and *Archaea*. The remaining networks, often featuring few domains with broad taxonomic distribution and poor characterization, were not amenable to our ‘guilty-by-association’ interpretative approach.

### Transitivity clustering

**Figure 3:**
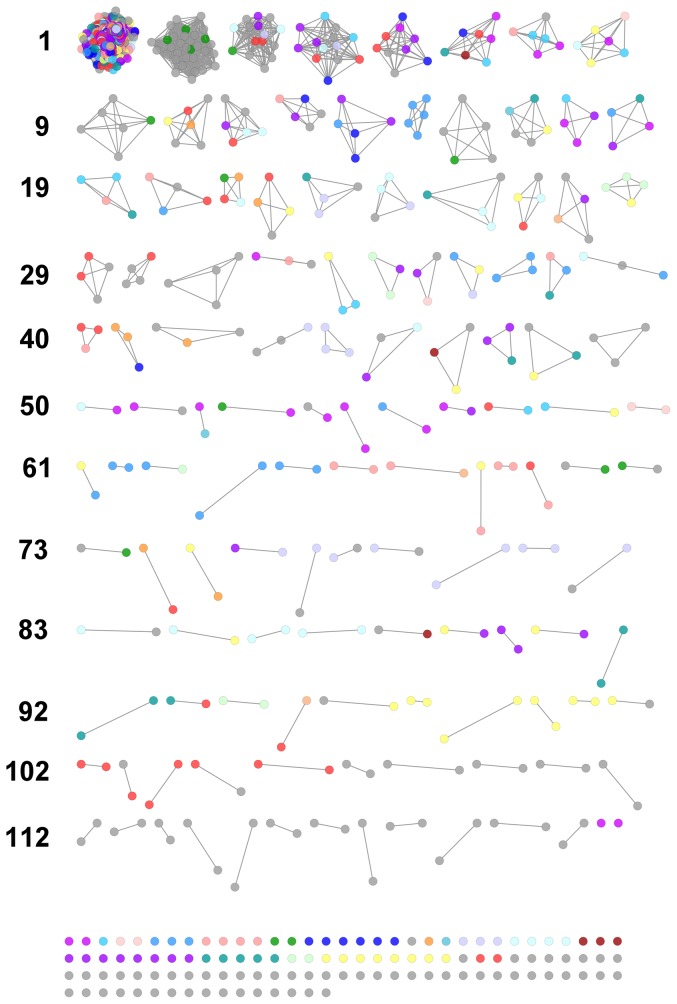
Transitivity clusters derived from correlations of Pfam abundances across selected GOS metagenomes (unstandardized data). Edge-weights (correlations) determine the cost of adding or removing edges during clustering. We observed clusters with domains linked to photobiology; oligotrophic adaptations; DNA maintenance and repair; and iron supply. Node colors represent functional categories; refer to **Figure S7** for description. See text and **Tables 3** **,** **4** **, and** **5** for detailed descriptions.

**Figure 4:**
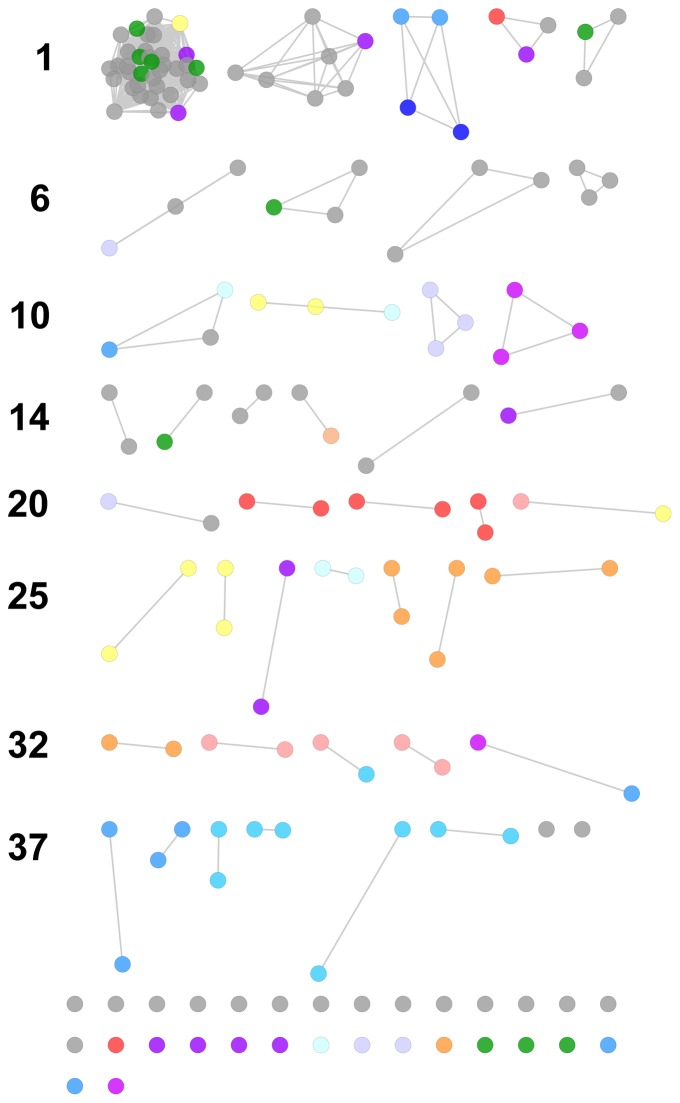
Transitivity clusters derived from correlative associations of Pfam domains across GOS metagenomes (standardized data). Edge-weights (correlations) determine the cost of adding or removing edges during clustering. The largest cluster contained DUFs and domains linked to photobiology (**Table S5**). Node colors represent functional categories; refer to **Figure S7** for description. See text for detailed descriptions.

Associations between DUFs in highly-enmeshed regions are difficult to evaluate by visual inspection. Transitivity clustering [25] offers a means to detect interconnected substructures in these regions, isolating them by adding and removing edges against a cost function (in this case derived from correlation strength). Our application of this method produced 49 clique-like transitivity clusters (TCs) with 3 or more members from the UM (**Figure 3**; see **Figure S5** for node labels) and 13 such clusters from the SM (**Figure 4** see **Figure S6** for node labels).

Much of the highly-enmeshed core of the UM-derived LCC remained in a single cluster (**Figure 3**: TC1, n = 464) which included 30 DUFs. The density and functional diversity of this region prevented speculation on DUF function. However, domains with known and related functions were clustered, including the urea and phosphonate metabolic components observed above (**Figure 3**: TC40, TC44; **Figure 4**: TC12, TC23). Further, the DNA mismatch repair domains, MutS I–V (**Figure 3**: TC14; **Figure 4**: TC37), which were initially obscured within enmeshed network regions, were clustered.

#### Photobiology

We observed prominent transitivity clusters with photobiologically relevant domain membership. A UM-derived cluster (**Figure 3**: TC2; **Table S7**) contained most domains from the photobiological region of the original network (**Figure 1**, Box 2). The membership of the largest SM-derived cluster (**Figure 4**: TC1; **Table S8**) was similar and included 28 DUFs, five photobiological domains, two cobalamin metabolism domains, and a fructosamine kinase domain.

Notably, several photobiologically relevant domains occurred in separate clusters. In the UM-derived clusters, a photosystem I reaction center domain was clustered with five DUFs, while a domain that has been implicated in the assembly and stability of photosystem I complex in chloroplasts was clustered with four DUFs (**Figure 3**: TC9 and TC15 resp.). Further, a photosystem I reaction center domain (PsaL) was clustered with DUF2839 (UM) and DUF1824 (SM). Lastly, Photo_RC and DUF1825 were associated in an UM-derived cluster (TC73) while an SM-derived cluster also included DUF3110 (TC5). These DUFs have distinct taxonomic distributions, which may partially account for their clustering (see Taxonomic perspectives, below). Cobalamin synthesis domains, which may participate in pigment synthesis, were clustered with the septum formation inhibitor domain, MinC_C. MinC_C shares a Pfam architecture with an amidase domain involved in cobalamin synthesis (CbiA) and was clustered with a domain which catalyzes the conversion of cobalamin into its coenzyme form; DUF3531 which occurs primarily in the *Cyanobacteria* and *Viridiplantae*; and DUF3104 which occurs primarily in the *Cyanobacteria* (**Figure 3**: TC27).

Several UM-derived clusters, discussed below, appeared to have a common functional theme, which suggested putative roles for the DUFs they contain.

#### Nutrient-limitation

We observed three UM-derived clusters (**Table 3**) featuring domains with special relevance in nutrient-limited environments, including capacities to flexibly metabolize nutrients, repress costly biosynthetic pathways, and cope with metabolic stress. Indeed, many marine microbes are adapted to low or intermittent nutrient availability and such adaptations are reflected in their genomes [26], [27]. A cluster comprising 13 members (**Figure 3**: TC4) included domains involved in molybdenum cofactor biosynthesis; nitrate assimilation and nitrate inducible dehydrogenase activity; phosphatase activity; oxygenation of nitrogen, sulfur, phosphorus and selenium atoms in xenobiotics; carbon-sulfur and carbon-nitrogen bond chemistry; and a chorismate mutase domain, whose activity promotes phenylalanine and tyrosine biosynthesis at the expense of tryptophan synthesis. Minimizing the synthesis of biochemically costly amino acids such as tryptophan [28] is a viable strategy to promote ecological competitiveness in oligotrophic conditions. Continuing this theme, a cluster including DUF3047 and DUF2155 (**Figure 3**: TC12) contained domains that catalyze the cleavage and cannibalization of nitrogen-bearing components from polyamines and purines. Lastly, a four-membered cluster (**Figure 3**: TC19) contained an organic solvent tolerance protein; a regulator of the acetate and glycerol operons and glyoxalate shunt; a domain involved in monitoring cellular nitrogen levels, the nitrogen stress response, and with possible roles in iron metabolism; and a fatty acid degradation regulatory domain, also found in regulators of sugar biosynthesis operons and iclR activators. These associations suggest that DUFs in these clusters may contribute to metabolic adaptations to oligotrophic conditions.

**Table 3:** Pfam domains contained in transitivity clusters putatively linked to nutrient-limitation (unstandardized data).

Cluster	Category	Pfam ID	Pfam Comment (abridged)
TC4	AA	Alliinase_C	Allicin is a thiosulphinate that gives rise to dithiines, allyl sulphides and ajoenes, the three groups of active compounds in Allium species. Allicin is synthesised from sulfoxide cysteine derivatives by alliinase, whose C-S lyase activity cleaves C(beta)-S(gamma) bonds. It is thought that this enzyme forms part of a primitive plant defence system.
		CM_2	Chorismate mutase catalyses the conversion of chorismate to prephenate in the pathway of tyrosine and phenylalanine biosynthesis. This enzyme is negatively regulated by tyrosine, tryptophan and phenylalanine.
	CoE	MoaC	Members of this family are involved in molybdenum cofactor biosynthesis. However their molecular function is not known.
		Mob_synth_C	This region contains two iron-sulphur (3Fe-4S) binding sites.
		ThiS	ThiS (thiaminS) is a 66 aa protein involved in sulphur transfer. Thiocarboxylate is formed at the last G in the activation process. Sulphur is transferred from ThiI to ThiS in a reaction catalysed by IscS. MoaD, Swiss:P30748 a protein involved sulphur transfer in molybdopterin synthesis, is about the same length and shows limited sequence similarity to ThiS.
	E	FdhD-NarQ	Nitrate assimilation protein, NarQ, and FdhD are required for formate dehydrogenase activity.
	Ion	FMO-like	This family includes FMO proteins, cyclohexanone monooxygenase Swiss:P12015, and Swiss:Q10532.
	NA	DUF3108	This bacterial family of proteins has no known function.
		DUF328	Members of this family are functionally uncharacterised. They are about 250 amino acids in length.
		DUF3501	This family of proteins is functionally uncharacterised. This protein is found in bacteria and archaea.
	Nuc	Ureidogly_hydro	Ureidoglycolate hydrolase carried out the third step in the degradation of allantoin.
	Transcr	NIF	This family contains a number of NLI interacting factor isoforms and also N-terminal regions of RNA polymerase II CTC phosphatase and FCP1 serine phosphatase. This region has been identified as the minimal phosphatase domain.
		Sigma70_ner	The domain is found in the primary vegetative sigma factor. The function of this domain is unclear and can be removed without loss of function.
TC12	NA	DUF2155	This domain, found in various hypothetical prokaryotic proteins, has no known function.
		DUF3047	This bacterial family of proteins has no known function.
	Nuc	Allantoicase	These proteins allow the use of purines as secondary nitrogen sources in nitrogen-limiting conditions.
	PostModChaps	DS	Eukaryotic initiation factor 5A (eIF-5A) contains an unusual amino acid, hypusine. The first step in the post-translational formation of hypusine is catalysed by the enzyme deoxyhypusine synthase (DS). The modified version of eIF-5A, and DS, are required for eukaryotic cell proliferation.
	Sig	HPP	These proteins are integral membrane proteins with four transmembrane spanning helices. The most conserved region of the alignment is a motif HPP. The function of these proteins is uncertain but they may be transporters.
TC19	CWME	OstA_C	Family involved in organic solvent tolerance in bacteria.
	PostModChaps	GlnD_UR_UTase	This is a family of bifunctional uridylyl-removing enzymes/uridylyltransferases (UR/UTases, GlnD) that are responsible for the modification of the regulatory protein P-II, or GlnB. In response to nitrogen limitation, these transferases catalyse the uridylylation of the PII protein, which in turn stimulates deadenylylation of glutamine synthetase (GlnA). Moreover, uridylylated PII can act together with NtrB and NtrC to increase transcription of genes in the sigma54 regulon, which include glnA and other nitrogen-level controlled genes. It has also been suggested that the product of the glnD gene is involved in other physiological functions such as control of iron metabolism in certain species.
	Transcr	IclR	This family of bacterial transcriptional regulators includes the glycerol operon regulatory protein and acetate operon repressor both of which are members of the iclR family. However this family covers the C-terminal region that may bind to the regulatory substrate (unpublished observation, Bateman A.).
		FCD	This domain is the C-terminal ligand binding domain of many members of the GntR family. This domain probably binds to a range of effector molecules that regulate the transcription of genes through the action of the N-terminal DNA-binding domain. This domain is found in Swiss:P45427 and Swiss:P31460 that are regulators of sugar biosynthesis operons.

Refer to Table 1, footnote for list of abbreviations.

#### DNA metabolism and repair

Two UM-derived clusters were characterized by DNA metabolism and maintenance domains (**Table 4**). The microbial communities of epipelagic waters face sustained solar irradiation, which causes DNA damage [29]. It is likely the following clusters reflect adaptations to cope with this environmental threat. A cluster of 9 nodes (**Figure 3**: TC5) included amino acid dehydrogenase domains; domains involved in the synthesis of purines and aromatic amino acids; domains involved in DNA synthesis and repair; and a tRNA synthetase domain. Milligan et al.'s in vitro finding on the ability of several amino acids to repair oxidative DNA damage by reducing guanyl radicals [30] may offer some insight into the presence of amino acid dehydrogenases in this cluster: epipelagic microbes may similarly couple amino acid oxidation and DNA radical deactivation. Another cluster (**Figure 3**: TC39) comprised DUF836, classified as a glutaredoxin-like domain; an inorganic pyrophosphatase domain; and a domain involved in recombination, repair of double strand DNA breaks, and resistance to irradiative and chemical DNA-damage. As a glutaredoxin-like domain, DUF836 may participate both in DNA metabolism through glutathione-dependent synthesis of deoxyribonucleotides and in antioxidant defense [31]. Glutaredoxins may also serve in the assembly and transfer of iron/sulfur complexes [32] and thus may have particular importance in safeguarding DNA integrity and metabolism in iron-limited marine waters.

**Table 4:** Pfam domains contained in transitivity clusters putatively linked to DNA maintenance and repair (unstandardized data).

Cluster	Category	Pfam ID	Pfam Comment
TC5	AA	ELFV_dehydrog_N	–
		Pro_dh	–
	CoE	DHFR_1	–
		ApbE	This prokaryotic family of lipoproteins are related to ApbE from Salmonella typhimurium. ApbE is involved in thiamine synthesis. More specifically is may be involved in the conversion of aminoimidazole ribotide (AIR) to 4-amino-5-hydroxymethyl-2-methyl pyrimidine (HMP).
	NA	DUF1800	This is a family of large bacterial proteins of unknown function.
		DUF177	–
	Nuc	ASL_C	This domain is found at the C-terminus of adenylosuccinate lyase(ASL; PurB in E. coli). It has been identified in bacteria, eukaryotes and archaea and is found together with the lyase domain Pfam:PF00206. ASL catalyses the cleavage of succinylaminoimidazole carboxamide ribotide to aminoimidazole carboxamide ribotide and fumarate and the cleavage of adenylosuccinate to adenylate and fumarate.
		Thymidylat_synt	Swiss:P28176 is not included as a member of this family, Although annotated as such there is no significant sequence similarity to other members.
	TransR	tRNA-synt_1c_C	Other tRNA synthetase sub-families are too dissimilar to be included. This family includes only glutamyl and glutaminyl tRNA synthetases. In some organisms, a single glutamyl-tRNA synthetase aminoacylates both tRNA(Glu) and tRNA(Gln).
TC39	E	Pyrophosphatase	–
	NA	DUF836	–
	RRR	Exonuc_V_gamma	The Exodeoxyribonuclease V enzyme is a multi-subunit enzyme comprised of the proteins RecB, RecC (this family) and RecD. This enzyme plays an important role in homologous genetic recombination, repair of double strand DNA breaks resistance to UV irradiation and chemical DNA-damage. The enzyme (EC:3.1.11.5) catalyses ssDNA or dsDNA-dependent ATP hydrolysis, hydrolysis of ssDNA or dsDNA and unwinding of dsDNA. This family consists of two AAA domains.

Refer to Table 1, footnote for list of abbreviations.

#### Iron-limitation

Seawater iron concentrations have previously been linked to iron metabolism genes in GOS [33], and the importance of iron supply may offer insight into two further clusters (**Table 5**). A four-membered cluster (**Figure 3**: TC23) contained DUF255; domains of an iron/manganese superoxide dismutase; and the Gram-negative bacterial TonB domain. TonB works in conjunction with outer membrane transport proteins in the active uptake of siderophore-bound iron (II) and cyanocobalamin. The ability of the TonB system to divert the proton motive force in aid of active iron transport is likely to be an asset in the typically iron-limited marine water column. Close association of this iron (II) supply-line to iron-dependent superoxide dismutases may be due, once again, to the threat of radicals generated in the irradiated water column. Another association of iron transport and iron-dependent enzyme domains was observed in a cluster (**Figure 3**: TC24) containing DUF58; a pair of pyruvate oxidoreductase domains; and a periplasmic binding protein domain often involved in iron transport. Pyruvate oxidoreductase has multiple iron/sulfur clusters and participates in the metabolism of short carboxylic acids as well as the carbon-fixing, reductive carboxylate cycle.

**Table 5:** Pfam domains contained in transitivity clusters putatively linked to iron supply and utilization (unstandardized data).

Cluster	Category	Pfam ID	Pfam Comment
TC23	CWME	TonB	–
	Ion	Sod_Fe_N	superoxide dismutases (SODs) catalyse the conversion of superoxide radicals to hydrogen peroxide and molecular oxygen. Three evolutionarily distinct families of SODs are known, of which the Mn/Fe-binding family is one.
		Sod_Fe_C	superoxide dismutases (SODs) catalyse the conversion of superoxide radicals to hydrogen peroxide and molecular oxygen. Three evolutionarily distinct families of SODs are known, of which the Mn/Fe-binding family is one.
	NA	DUF255	–
TC24	E	POR	This family includes a region of the large protein pyruvate-flavodoxin oxidoreductase and the whole pyruvate ferredoxin oxidoreductase gamma subunit protein. It is not known whether the gamma subunit has a catalytic or regulatory role. Pyruvate oxidoreductase (POR) catalyses the final step in the fermentation of carbohydrates in anaerobic microorganisms. This involves the oxidative decarboxylation of pyruvate with the participation of thiamine followed by the transfer of an acetyl moiety to coenzyme A for the synthesis of acetyl-CoA. The family also includes pyruvate flavodoxin oxidoreductase as encoded by the nifJ gene in cyanobacterium which is required for growth on molecular nitrogen when iron is limited.
		POR_N	This family includes the N terminal structural domain of the pyruvate ferredoxin oxidoreductase. This domain binds thiamine diphosphate, and along with domains II and IV, is involved in inter subunit contacts. The family also includes pyruvate flavodoxin oxidoreductase as encoded by the nifJ gene in cyanobacterium which is required for growth on molecular nitrogen when iron is limited.
	Ion	Peripla_BP_2	“This family includes bacterial periplasmic binding proteins. Several of which are involved in iron transport.”
	NA	DUF58	This family of prokaryotic proteins have no known function. Swiss:P71138 a protein of unknown function in the family has been misannotated as alpha-dextrin 6-glucanohydrolase.

Refer to Table 1, footnote for list of abbreviations.

#### Miscellaneous clusters

We observed several UM-derived clusters that we could not place in a larger interpretive framework, but which included DUFs in a noteworthy functional context (**Table S9**). For example, DUF484 and DUF2066 were clustered with domains involved in sodium translocation linked to amino acid transport and redox as well as a ligase that initiates the glutathione biosynthesis pathway (**Figure 3**: TC11). The glutathione pathway is implicated in a wide range of cellular functions, including amino acid transport, and has been specifically linked to sodium-dependent transport in eukaryotic systems [34], [35]. Next, DUF37, annotated as a haemolytic domain in Pfam v26, was clustered with a number of domains implicated in transcription and ribosomal function (**Figure 3**: TC7). This cluster is similar to a network derived from the SM (**Figure 2**, ii) and a spoke of the UM (**Figure 1**, Box 3; **Table 2**). Another cluster was characterized by domains capable of phosphorylation (**Figure 3**: TC10) and contained a DUF (DUF894) reclassified as a transmembrane secretion effector in a major facilitator superfamily (MFS_3); several kinase domains including a DUF (DUF227) reclassified as a kinase targeting insect hormones (EcKinase); and a selenium transferase domain. Phosphorylation plays a key role in selenocysteine formation through selenophosphate in Archaea and Eukarya. A pair of proline metabolism domains was clustered with DUF525 and DUF461 (**Figure 3**: TC29). Lastly, DUF192, putatively involved with extracellular sugar processing, was clustered with an isomerase domain, which functions in both the pentose phosphate pathway and the Calvin cycle, and a Type I restriction modification DNA specificity domain (**Figure 3**: TC46). This latter domain targets and either degrades DNA foreign to bacterial cells (such as viral DNA) or methylates DNA. This cluster may reflect the coupling of viral defense mechanisms to sugar scavenging – a possible adaptation to capitalize on the ‘spoils of war’ in resource limited environments. Despite their isolation, the above clusters offer interesting perspectives on the involvement of DUFs in epipelagic community metabolism.

Several SM-derived clusters may also grant interesting perspectives on DUF co-occurrence. An Mg^2+^-dependent acid phosphatase involved in the biosynthesis of several cofactors including cobalamin and heme was clustered with six DUFs (**Figure 4**: TC2). These DUFs are prevalent in the *Cyanobacteria* (see below) with very low representation in the *Proteobacteria*, a restriction that may account for their clustering. However, domains in several smaller clusters showed dissimilar taxonomic distributions. These included a cluster (**Figure 4**: TC6) comprising a voltage-gated chloride channel, DUF2930, and DUF2214, the latter predicted to be a membrane protein. Pairs of domains with dissimilar taxonomic distributions were also observed: DUF3531 and DUF3641; a cobalamin-5-phosphate synthase domain (CobS) and DUF3727; a septum formation inhibitor (MinC_C) and DUF3119; DUF2010, reclassified as a mitochondrial PGP phosphatase, and DUF1823; and a divalent ion tolerance protein (CutA1) and DUF92. Comments on other transitivity clusters are available in the supplementary **Material S1**.

### Taxonomic perspectives

The taxonomic distribution of Pfam domains, particularly those present in the highly abundant marine *Cyanobacteria*, will inevitably influence their association and condition any interpretation. We retrieved the taxonomic distribution of the DUF families analyzed above from the Pfam web-portal to qualitatively contextualize the observed associations. However, quantifying the degree to which the taxonomic make-up of microbial communities confounds functional associations in metagenomic samples is a non-trivial task. Such assertions are contingent on the taxonomy and functional annotation of the current genome collection, which is unlikely to reflect the true *in situ* diversity. Further, correct assignment of sequencing reads to known taxa is often problematic. Thus, the taxonomic distributions presented below are intended to provide a tentative context to advise hypothesis generation (as demonstrated above) and are not intended as a basis for phylogenomic profiling.

In the UM-derived clusters (**Figure 3**, **Table 6**), four phyla contained greater than 5% of DUF instances, namely the *Proteobacteria*, *Firmicutes*, *Actinobacteria*, and *Cyanobacteria*. The DUFs of the largest UM-derived transitivity cluster (TC1) had similar distributions to the whole collection, while DUFs whose instances were concentrated in cyanobacterial genomes dominated the second cluster (∼55%). The proteobacterial proportion of this latter cluster was dominated by the *Gammaproteobacteria* (∼56%) and a proportion of *Alphaproteobacteria* (∼23%). The genera *Rhodobacterales* and *Rhodospirillales*, known to possess proteorhodopsin genes encoding light-powered proton pumps, were present in the alphaproteobacterial division. As reviewed by DeLong and Béjà [36], there is increasing evidence that proteorhodopsin pumps and light-powered heterotrophy are more broadly distributed in epipelagic bacteria than previously thought. In fact, proteorhodopsin pumps have recently been identified in marine eukaryotes [37] suggesting the limits of their occurrence in marine microbes has yet to be fully established. This suggests that the correlation of these DUFs may not be entirely due to their restriction to photoautotrophs. Indeed, DUFs with distributions more restricted to photoautotrophs were clustered separately. For example, less than a percentage of organisms bearing DUFs from TC9 were *Proteobacteria* and those from TC15 were almost exclusively *Cyanobacteria* (∼95%). Other clusters with conspicuous taxonomic restrictions included TC31, with domains prevalent in actinobacterial genomes, and TC43, with domains prevalent in bacterial, fungal, and plant genomes.

**Table 6:** Phylum-level* taxonomic distribution of DUFs in selected transitivity clusters (unstandardized data).

Sample	n(DUF)	Phylum	Instances	% of total instances
**Overall**	225	*Proteobacteria*	73177	47.83
		*Firmicutes*	26904	17.59
		*Actinobacteria*	15128	9.89
		*Cyanobacteria*	7798	5.10
**TC1**	30	*Proteobacteria*	21877	46.11
		*Firmicutes*	10783	22.73
		*Actinobacteria*	4646	9.79
**TC2**	28	*Cyanobacteria*	2206	54.48
		*Proteobacteria*	820	20.25
		*Viridiplantae*	588	14.52
		*Firmicutes*	240	5.93
**TC3**	8	*Proteobacteria*	2199	77.32
		*Actinobacteria*	202	7.10
		*Fungi*	145	5.10
**TC4**	3	*Proteobacteria*	1590	62.62
		*Firmicutes*	363	14.30
		*Bacteroidetes*	289	11.38
		*Actinobacteria*	160	6.30
**TC9**	5	*Cyanobacteria*	362	64.07
		*Viridiplantae*	155	27.43
**TC10**	3	*Proteobacteria*	2027	35.42
		*Metazoa*	1140	19.92
		*Firmicutes*	1019	17.81
		*Actinobacteria*	971	16.97
**TC11**	2	*Proteobacteria*	1021	98.74
**TC12**	2	*Proteobacteria*	317	94.63
**TC15**	4	*Cyanobacteria*	266	92.68
		*Viridiplantae*	15	5.23
**TC16**	2	*Proteobacteria*	355	64.20
		*Actinobacteria*	163	29.48
		*Metazoa*	30	5.42
**TC29**	2	*Proteobacteria*	1614	79.16
		*Actinobacteria*	120	5.89
**TC30**	3	*Proteobacteria*	1363	67.24
		*Actinobacteria*	303	14.95
		*Cyanobacteria*	126	6.22
**TC31**	4	*Actinobacteria*	1708	71.73
		*Proteobacteria*	218	9.16
		*Fungi*	159	6.68
		*Firmicutes*	143	6.01
**TC49**	3	*Proteobacteria*	1480	76.21
		*Firmicutes*	202	10.40
		*Bacteroidetes*	193	9.94

* Only Phyla with >5% of DUF instances are shown.

The DUFs in SM-derived clusters (**Figure 4**, **Table 7**) were predominately found in cyanobacterial genomes (≥50% of all occurrences). Exceptions included TC6 and TC7, which contained DUFs comparably distributed between proteobacterial and cyanobacterial genomes, as well as TC14 which featured DUFs distributed in the *Fungi*, *Firmicutes*, and *Cyanobacteria*.

**Table 7:** Phylum-level* taxonomic distribution of DUFs in selected transitivity clusters (standardized data).

Sample	n(DUF)	Phylum	Instances	% of total instances
**Overall**	75	*Proteobacteria*	5575	32.31
		*Cyanobacteria*	4845	28.08
		*Firmicutes*	1946	11.28
		*Viridiplantae*	1505	8.72
		*Actinobacteria*	1026	5.95
**TC1**	28	*Cyanobacteria*	2290	54.48
		*Proteobacteria*	760	18.08
		*Viridiplantae*	600	14.28
		*Firmicutes*	244	5.81
**TC2**	6	*Cyanobacteria*	338	66.14
		*Viridiplantae*	73	14.29
		*Actinobacteria*	52	10.18
		*Firmicutes*	29	5.68
**TC5**	2	*Cyanobacteria*	125	65.79
		*dsDNAvirusesnoRNAstage*	30	15.79
		*Viridiplantae*	24	12.63
**TC6**	2	*Proteobacteria*	109	37.59
		*Cyanobacteria*	91	31.38
		*Viridiplantae*	77	26.55
**TC7**	2	*Proteobacteria*	109	37.59
		*Cyanobacteria*	91	31.38
		*Viridiplantae*	77	26.55
**TC8**	3	*Cyanobacteria*	199	70.32
		*Viridiplantae*	41	14.49
		*Proteobacteria*	19	6.71
**TC14**	2	*Fungi*	89	28.34
		*Firmicutes*	69	21.97
		*Cyanobacteria*	63	20.06
		*Viridiplantae*	34	10.83
		*Proteobacteria*	24	7.64
**TC16**	2	*Cyanobacteria*	121	51.05
		*Viridiplantae*	71	29.96
		*Chlorobi*	24	10.13
**TC18**	2	*Cyanobacteria*	113	39.37
		*Viridiplantae*	53	18.47
		*Proteobacteria*	42	14.63
		*Firmicutes*	30	10.45
		*Bacteroidetes*	16	5.57

* Only Phyla with >5% of DUF instances are shown.

### Establishing benchmarks

Assigning the associations discussed above with meaningful measures of confidence is an immediate concern. False positives may lead experimentalists in fruitless directions, while false negatives may limit functional discovery. Similar difficulties are encountered when attempting to benchmark protein interaction networks and attempts to minimize them rely on the reproducibility of interactions across datasets or the use of well-characterized model systems or gold standards [38].

Several aspects of metagenomic data currently hinder the construction of such benchmarks. Firstly, metagenomes are likely to contain genome fragments from organisms with no metabolically well-characterized counterparts. This greatly weakens the credibility of model-systems or –organisms as gold standards. Secondly, studies on the scale of the GOS expedition are, presently, difficult to replicate. The increasing collection of marine metagenomes, fed by initiatives such as TARA Oceans [39], as well as the growing genome collection driven by programs such as the Genomic Encyclopedia of *Bacteria* and *Archaea* [40], may soon offer the opportunity to construct such standards. However, experimental confirmation or falsification of our assertions by bench scientists is perhaps the most conclusive basis for evaluation. In the spirit of initiatives such as the Computational Bridge to Experiments (ComBrEx) [41], groups with the infrastructure and expertise to test *in silico* predictions *in vitro* may, en masse, provide a degree of confidence estimation for studies similar to ours. DUFs which have been described as at least partially characterized in subsequent releases of Pfam may anticipate such estimation. Relative to Pfam v24, we noted 28 DUF families present in our UM and SM datasets which have been renamed or merged into existing families in Pfam v26 (**Table S10**). In the UM-derived data, eight of these DUFs were not clustered while a further eight occurred in the highly-enmeshed TC1 (**Figure 3**). We were thus unable to compare these updates to our results. However, the updated descriptions of six of the remaining DUFs – DUF989, DUF403, DUF404, DUF407, DUF227, and DUF1008 – reflect their clustering in this analysis. For example, a heme-binding domain towards the C-terminus of DUF989, now merged with the Cytochrome-C family, is clustered with families such as CobT, involved in cobalamin synthesis. The chemical similarity of the corrin and porphyrin rings found in cobalamin and heme, respectively, may provide a basis for the co-occurrence of these domains. The remaining DUFs have been compared to their updated descriptions in the sections above.

While the novelty and scale of metagenomic datasets present obstacles in constructing gold standards, these factors are assets in inferring functionality through intra-ecosystemic domain covariation. We attempted to limit false assertions by using conservative detection and correlation thresholds coupled with knowledge-guided curation of our results. These approaches were used to gauge if domain associations offered plausible insights into DUF functionality in an epipelagic setting, and noteworthy associations were discussed above.

### Conclusion

We observed that intercorrelation of protein domain sequences across intra-ecosystem metagenomic datasets can provide perspectives on the potential roles of domains of unknown function. Naturally, even strong correlation across metagenomic datasets cannot provide direct functional annotations, as numerous factors may account for domain covariance in natural systems. However, critically evaluating strongly correlated domains with knowledge-level resources can provide an interpretive context to complement more targeted efforts in DUF characterization. The observed association of domains involved in microbial phosphonate metabolism, urea metabolism, and other ecologically relevant capacities encourage this approach. Of the 225 and 75 DUFs retained following correlation analysis in the UM and SM datasets respectively, we detected 94 (UM) and 48 (SM) DUFs with connectivity biased towards a single metabolic category. Further, the results above list 73 DUFs from the UM-derived and 41 DUFs from SM-derived networks and transitivity clusters whose associations may reflect ecological features of the marine epipelagic zone. While these results represent only a fraction of the DUFs detected in the GOS metagenomes, this analysis is an initial step in using ecogenomic variation to assist functional discovery. The opportunity to routinely perform such exploratory analyses and establish quantitative benchmarks is emerging as data from large-scale metagenomic, metatranscriptomic, and metaproteomic sampling campaigns becomes publically available. The perspectives that can be derived from this data will almost certainly forward efforts to characterize DUFs where homology-based approaches cannot.

## Materials and Methods

### Detecting domains of unknown function

A collection of 10,133,846 unassembled reads from the Global Ocean Sampling expedition metagenomes GS000a-GS023, GS025-GS051, GS108a-GS117b, GS119-GS123, GS148-GS149, and MOVE858 [16] were downloaded from the CAMERA web-portal [42] and queried against all hidden Markov models (HMMs) present in the Pfam 24 database using the HMMER3 software (version 3.0b3). Hits were deemed significant if their domain independent E-value was less than or equal to 1e-3, their bias composition correction was at least an order of magnitude less than their full score, the length of the query alignment was at least 20% of the query length, and the model alignment was at least 20% of the HMM length. Results were stored in a relational database and cross-tabulated into a “site×Pfam” matrix, wherein the abundance of each Pfam at a given site was enumerated. Pfams assigned to COG functional metabolic categories available from the integrated microbial genomes (IMG) system [43], as well as an additional category for photobiologically active domains (**Table S1**), were used in further analyses.

### Correlation analysis and network visualization

The site×Pfam matrix described above was imported into the R statistical computing environment (http://cran.r-project.org/). Pfam categories listed in more than one metabolic category were removed. Distributions of total, non-zero abundances across Pfam categories and sites were used to advise data preparation. Categories with less than 20 non-zero abundances across the 80 sites analyzed and sites with less than 1,000 non-zero abundances across the 3,587 Pfam categories analyzed were removed. A copy of the resulting matrix was subject to row standardization, whereby the Pfam abundances across a given site (row) were divided by the maximum Pfam abundance of that site. The Spearman's rank correlation of Pfam categories in both matrices was determined using the *rcorr()* function from the R package Hmisc. Pfam categories with no correlations greater than a rho of 0.80 and with a P-value less than a Bonferroni corrected cut-off of ∼1×10^−6^ were removed. DUFs that showed correlations biased towards Pfams in a single metabolic category were noted. Bias was declared when the number of correlations of a DUF to Pfams in a given category was at least double that of correlations to any other category.

The igraph R package [44] was used to create an adjacency matrix from these correlation results. This was imported into Cytoscape [45] for visualization. Network vertices, each corresponding to a Pfam category, were connected by an edge if their correlation satisfied the thresholds stated above. The Spearman's rho statistic provided weights in an edge-weighted, spring-embedded visualization. Pfam categories were color-coded according to their assigned metabolic category.

### Network exploration

Networks were manually inspected for distinct topological features, particularly those where DUFs were associated with characterized Pfams from a narrow range of COG categories. The domains comprising these features were investigated further by retrieving descriptions from Pfam 26 via its webportal (www.pfam.sanger.ac.uk) and further literature where appropriate.

The TransClust [25], [46] algorithm was run from the Cytoscape plugin, clusterMaker [47], to detect clique-like clusters. TransClust was run with a maximum sub-cluster size set to 50, a maximum time allowance of 2 seconds to execute each loop in the algorithm, and using edge weight (correlation) as an array source. The resulting clusters were visualized and evaluated as described above.

### Taxonomic annotation

The DUFs in several transitivity clusters were annotated with their collective taxonomic distribution. Distributions were retrieved from the Pfam website (www.pfam.sanger.ac.uk; 2012-02-10) and the results stored in a relational database. Only clusters with ≥4 members and ≥2 DUFs were examined and those with notable distributions were discussed.

## Supporting Information

Material S1 **Comments on selected transitivity clusters not discussed in text.**
(DOC)

Table S1 **Pfam 24 domains categorized as photobiologically relevant in this analysis.**
(DOC)

Table S2 **DUFs with correlative bias towards photobiologically relevant domains (unstandardized data).**
(DOC)

Table S3 **DUFs with correlative bias towards metabolic categories (standardized data).** Refer to **Table 1**, footnote for list of abbreviations.
(DOC)

Table S4 **Domains present in a network region characterized by photobiologically relevant domains (unstandardized data;** **Figure 1** **, Box 2).** Refer to **Table 1**, footnote for list of abbreviations.
(DOC)

Table S5 **Domains present in a network region bridging two enmeshed regions (unstandardized data;** **Figure 1** **, Box 4).** Refer to **Table 1**, footnote for list of abbreviations.
(DOC)

Table S6 **Domains present in the primary network generated from standardized Pfam abundances across GOS sites (** **Figure 2, i** **).** Refer to **Table 1**, footnote for list of abbreviations.
(DOC)

Table S7 **Pfam domains contained in the second largest transitivity cluster derived from unstandardized domain abundances (** **Figure 3** **: TC2).** Refer to **Table 1**, footnote for list of abbreviations.
(DOC)

Table S8 **Pfam domains contained in the largest transitivity cluster derived from standardized domain abundances (** **Figure 4** **: TC1).** Refer to **Table 1**, footnote for list of abbreviations.
(DOC)

Table S9 **Pfam domains contained in the selected transitivity clusters derived from unstandardized domain abundances (** **Figure 3** **).** Refer to **Table 1**, footnote for list of abbreviations.
(DOC)

Table S10 **DUFs with updated statuses in Pfam v26 and their location in transitivity clusters (TCs) from both the unstandardized (UM;** **Figure 3** **) and standardized (SM;** **Figure 4** **) datasets.**
(DOC)

Figure S1 **Histogram of correlation strength between unstandardized abundances of Pfam domains across GOS metagenomes.** The distribution's mean and standard deviation were ∼0.41 and ∼0.26 respectively. A normal distribution with equal mean and standard deviation is indicated by a blue contour.
(TIF)

Figure S2 **Histogram of correlation strength between abundances of Pfam domains across GOS metagenomes, standardized by site maxima.** The distribution's mean and standard deviation were ∼0.03 and ∼0.19 respectively. A normal distribution with equal mean and standard deviation is indicated by a blue contour.
(TIF)

Figure S3 **High resolution, unannotated version of** **Figure 1** **with individual node labels.**
(TIF)

Figure S4 **High resolution, unannotated version of** **Figure 2** **with individual node labels.**
(TIF)

Figure S5 **High resolution, unannotated version of** **Figure 3** **with individual node labels.**
(TIF)

Figure S6 **High resolution, unannotated version of** **Figure 4** **with individual node labels.**
(TIF)

Figure S7 **Color key for network nodes.**
(TIF)
