# Supplementary material for: Ecogenomic Perspectives on Domains of Unknown Function: Correlation-Based Exploration of Marine Metagenomes
Source: PLoS One. 2013 Mar 14;8(3):e50869. doi: 10.1371/journal.pone.0050869 (PMC3597751; doi:10.1371/journal.pone.0050869)
Supplement: Material S1 — Comments on selected transitivity clusters not discussed in text. (DOC) [file pone.0050869.s001.doc]

# Ecogenomic Perspectives on Domains of Unknown Function: Correlation-based Exploration of Marine Metagenomes

**Supplementary Information**

**Buttigieg et al.**

**Further results: Network exploration**

A peripheral region of the network derived from the UM, comprised a domain responsible for membrane hydrolysis during sporulation (SpoIID), a putative Mg^2+^ transporter (MgtE), an α-linked glucan-specific glycoside hydrolase (Glyco_hydro_77), and a chorismate mutase domain involved in the synthesis of phenylalanine and tyrosine at the expense of tryptophan (CM_1) was observed. These domains may be associated due to shared functionality in sporulation and germination. In this scenario, SpoIID would act in reshaping cellular membranes while MgtE expression would modulate internal Mg^2+^ concentrations, long implicated in bacterial sporulation [1]. The involvement of aromatic amino acids and the reactions of the shikimate pathway in sporulation and germination has been noted in both the *Bacteria* [2–4] and yeast [5]. The presence of the Glyco_hydro_77 domain in this association may be due to its role in liberating readily-metabolized sugars from large glucans such as α-amylose [6]. The nutrient-limited conditions that induce sporulation are likely to be characterized by a lack of simple carbohydrate molecules requiring degradation of polymers by enzymes such as amylases. Indeed, expression of α-amylase has been linked to sporulation in *Bacillus subtilis* [7] in the context of hierarchical usage of carbon sources [8].

## Further results: Transitivity Clustering

A cluster with 16 members, 8 of which were DUFs, contained a tellurite resistance domain (TerB); an electron transfer flavoprotein-ubiquinone oxidoreductase domain (ETF_QO); a phosphoenolpyruvate carboxykinase domain (PEPCK_ATP); a cobalamin biosynthesis protein domain (CobT); a coproporphyrinogen III oxidase domain (Coprogen_oxidas) involved in heme and chlorophyll synthesis; a sarcosine oxidative demethylase subunit domain (SoxD); and a domain implicated in photosystem I stabilization (BtpA).

A 10-membered cluster contained a methylpurine-DNA glycosylase domain (Pur_DNA_glyco) involved in base excision repair; a domain implicated in divalent ion tolerance (CutA1); and a glycoside hydrolase family (Glyco_hydro_77, discussed above). Another contained two DNA polymerase domains (DNA_pol_B_exo and DNA_pol_B), two exonuclease domains (DNA_pol_B_exo1 and Exonuc_X-T_C), a DNA methyltransferase domain (MethyltransfD12), a glutathione S-transferase domain (GST_C), and a domain found in fatty acid chain length determinant proteins and bacterial tyrosine kinases (Wzz).

A 7-membered cluster was observed and included a chromatin-associated protein domain (KTI12); a sigma-70 domain (Sigma70_r3); a phosphoribosyl-AMP cyclohydrolase (PRA-CH) involved in histadine biosynthesis; an RNA ribose methyltransferase substrate-binding domain (SpoU_sub_bind); an uncharacterized domain (NLPC_P60); a ribosomal protein S5 C-terminal domain (Ribosomal_S5_C); an MviN-like protein domain (MVIN) linked to virulence and motility in some bacteria.

A 6-membered cluster contained an alpha amylase domain (Alpha_amylase), a fructose-bisphosphate aldolase (F_bP_aldolase) domain, a subunit of ATP synthase (ATP_synt_A), a hydrolase domain (Isochorismatase), a negative regulator of heat-shock genes (HrcA), and a possible tRNA-methyltransferase domain (Nol1_Nop2_Fmu).

**5 membered clusters:**

Two ribonucleotide reductase domains (Ribonuc_red_sm, Ribonuc_red_lgC) were clustered with an oxygen-independent coproporphyrinogen-III oxidase (HemN_C); a cytosine-specific DNA methylase (DNA_methylase); and an extracellular sensory domain (CHASE2).

A domain needed for the export of envelope proteins (SecB) was clustered with an oligosaccharide biosynthesis protein (Alg14); a D-galactarate dehydratase / altronate hydrolase domain (GD_AH_C); an alpha/beta hydrolase of unknown function (DUF1057); and a predicted transcriptional regulator (DUF2083).

**4 membered clusters:**

An amidotransferase domain that promotes tRNA fidelity of glutamine translation (Glu-tRNAGln) was clustered with a putative thiamine biosynthetic enzyme (Thi4); a ribosomal protein domain (Ribosomal_L6); and a transcription elongation factor domain (GreA_GreB_N).

Another cluster comprised an aminoacyl-tRNA synthetase domain (Phe_tRNA-synt_N); a porphobilinogen deaminase (Porphobil_deamC); an acyltransferase involved in lipida A biosynthesis (LpxD); and an initiator of DNA replication (Bac_DnaA_C).

DUF971 was clustered with a glutamate fermentation domain (MAAL_C); a crossover junction endodeoxyribonuclease (RuvC); and a bacterial trigger factor protein domain (Trigger_C) with chaperone and isomerase activity.

A fatty acid desaturase domain (FA_desaturase) was clustered with a nitrogenase oxidoreductase domain (Oxidored_nitro); a lycopene cyclase domain (Lycopene_cycl) involved in carotenoid synthesis; and a metallopeptidase (Peptidase_M32).

An acyl-CoA thioesterase domain (Acyl_CoA_thio) was clustered with a proton-dependent oligopeptide transporter (PTR2); DUF179; and a glycoside hydrolase domain (Glyco_hydro_20).

A cytochrome b component of respiratory chain complex III (Cytochrom_B_N) was clustered with a transferase domain (Glyco_transf_8) involved in lipopolysaccharide and glycogen synthesis; a nickel-dependent cytochrome domain (Ni_hydr_CYTB), and DUF1467.

A glucokinase domain (Glucokinase) was clustered with a citrate isomerase (Aconitase_2_N); DUF482; and a regulatory domain of threonine dehydratase (Thr_dehydrat_C).

A flagellar motor switch protein (FliM) was clustered with a flagellar basal body-associated protein (FliL); a component of the flageller rotor (FliG_C); and an isomerase involved in pectin degradation and myo-inositol catabolism (KduI).

**3 membered clusters:**

A putative phosphatase (DUF442) was clustered with a leucyl/phenylalanyl-tRNA protein transferase (Leu_Phe_trans) and a uracil methyltransferase (tRNA_U5-meth_tr).

A domain found in aquaporins and transporters of small neutral solutes (MIP) was clustered with the DNA- and core-binding domains of the nitrogen-limitation sigma factor (Sigma54_DBD; Sigma54_CBD).

Bacterial flagellin N- and C-terminal helical regions (Flagellin_N; Flagellin_C) were clustered with a component of a Ca^2+^-sensitive G protein signaling cascade (Guanylate_cyc).

A carotenoid oxygenase (RPE65) was clustered with a 6-pyruvoyl tetrahydrobiopterin synthase domain (PTPS), involved in cofactor synthesis for aromatic amino acid hydroxylase activity, and DUF490.

A glutaredoxin-like domain (DUF836), possibly involved in the glutathione-dependent synthesis of deoxyribonucleotides, antioxidant defense, and iron-sulfur cluster transfer, was clustered with an inorganic pyrophosphatase domain (Pyrophosphatase) and the RecC domain of the DNA maintenance enzyme, exodeoxyribonuclease V (Exonuc_V_gamma).

Two myo-inositol-1-phosphate synthase domains (Inos-1-P_synth, NAD_binding_5) were clustered with a vitamin B12-dependent ribonucleotide reductase (Ribonuc_red_2_N).

A cobalamin-dependent, methylmalonyl-CoA mutase domain (MM_CoA_mutase) which initiates a radical conversion was clustered with DUF805 and DUF354. The former DUF predominately occur in bacterial genomes, while the latter is found predominately in archaeal genomes.

DUF952 and DUF924 were clustered with a domain involved in active sodium uptake (TrkH).

A molybdopterin guanine dinucleotide synthesis domain (MobB) was clustered with a cytochrome c domain (Cytochrom_C_2), characterized by heme c groups, and DUF3576.

Cobalamin-5-phosphate synthase (CobS) and a domain involved in the metabolism of riboflavin, porphyrin and chlorophyll (DBI_PRT also known as CobT) were clustered with an alanine dipeptidase (Peptidase_M15).

DUF779 was clustered with a penicillin-binding domain (PBP5_C) and a glycoside hydrolase family (Glyco_hydro_4).

DUF548, a putative SAM-dependent methyltransferase, was clustered with a putative bacterial lipoprotein (DUF940) and DUF3450.

**References**

1. Scribner H, Eisenstadt E, Silver S (1974) Magnesium transport in *Bacillus subtilis* W23 during growth and sporulation. J Bacteriol 117: 1224–1230.

2. Aronson JN, Wermus GR (1965) Effects of m-tyrosine on growth and sporulation of *Bacillus* species. J Bacteriol 90: 38–46.

3. Trach K, Hoch J a (1989) The *Bacillus subtilis* spo0B stage 0 sporulation operon encodes an essential GTP-binding protein. J Bacteriol 171: 1362–1371.

4. Ireland JAW, Hanna PC (2002) Amino acid- and purine ribonucleoside-induced germination of *Bacillus anthracis* sterne endospores: gerS mediates responses to aromatic ring structures. J Bacteriol 184: 1296–1303.

5. Lucchini G, Biraghi A, Carbone ML, de Scrilli A, Magni GE (1978) Effect of mutation in the aromatic amino acid pathway on sporulation of *Saccharomyces cerevisiae*. J Bacteriol 136: 55–62.

6. MacGregor E, Janeček Š, Svensson B (2001) Relationship of sequence and structure to specificity in the α-amylase family of enzymes. Biochim Biophys Acta 1546: 1–20.

7. Henkin TM, Grundy FJ, Nicholson WL, Chambliss GH (1991) Catabolite repression of  α amylase gene expression in *Bacillus subtilis* involves a trans-acting gene product homologous to the *Escherichia coli* lacl and galR repressors. Mol Microbiol 5: 575–584.

8. Görke B, Stülke J (2008) Carbon catabolite repression in bacteria: many ways to make the most out of nutrients. Nat Rev Microbiol 6: 613–624.
