# Supplementary material for: Ecogenomic Perspectives on Domains of Unknown Function: Correlation-Based Exploration of Marine Metagenomes
Source: PLoS One. 2013 Mar 14;8(3):e50869. doi: 10.1371/journal.pone.0050869 (PMC3597751; doi:10.1371/journal.pone.0050869)
Supplement: Table S1 — Pfam 24 domains categorized as photobiologically relevant in this analysis. (DOC) [file pone.0050869.s002.doc]

Table S1: Pfam 24 domains categorized as photobiologically relevant in this analysis

| **Pfam ID** | **Pfam accession** | **Description** |
| --- | --- | --- |
| Bac_chlorC | PF02043 | Bacteriochlorophyll C binding protein |
| BCHF | PF07284 | 2-vinyl bacteriochlorophyllide hydratase (BCHF) |
| BChl_A | PF02327 | Bacteriochlorophyll A protein |
| BLUF | PF04940 | Sensors of blue-light using FAD |
| BtpA | PF03437 | BtpA family |
| CytB6-F_Fe-S | PF08802 | Cytochrome B6-F complex Fe-S subunit |
| CytoC_RC | PF02276 | Photosynthetic reaction centre cytochrome C subunit |
| Cytochrome-c551 | PF10643 | Photosystem P840 reaction-centre cytochrome c-551 |
| DHC | PF09626 | Dihaem cytochrome c |
| DNA_photolyase | PF00875 | DNA photolyase |
| DPRP | PF04244 | Deoxyribodipyrimidine photo-lyase-related protein |
| FAD_binding_7 | PF03441 | FAD binding domain of DNA photolyase |
| Fe_bilin_red | PF05996 | Ferredoxin-dependent bilin reductase |
| Glyco_hydro_67C | PF07477 | Glycosyl hydrolase family 67 C-terminus |
| Glyco_hydro_67M | PF07488 | Glycosyl hydrolase family 67 middle domain |
| Glyco_hydro_67N | PF03648 | Glycosyl hydrolase family 67 N-terminus |
| HEAT_PBS | PF03130 | PBS lyase HEAT-like repeat |
| Lycopene_cycl | PF05834 | Lycopene cyclase protein |
| Mg_chelatase | PF01078 | Magnesium chelatase, subunit ChlI |
| MSP | PF01716 | Manganese-stabilising protein / photosystem II polypeptide |
| NdhL | PF10716 | NADH dehydrogenase transmembrane subunit |
| PCP | PF02429 | Peridinin-chlorophyll A binding protein |
| PetG | PF02529 | Cytochrome B6-F complex subunit 5 |
| Photo_RC | PF00124 | Photosynthetic reaction centre protein |
| PRCH | PF03967 | Photosynthetic reaction centre, H-chain N-terminal region |
| PsaA_PsaB | PF00223 | Photosystem I psaA/psaB protein |
| PsaD | PF02531 | PsaD |
| PsaL | PF02605 | Photosystem I reaction centre subunit XI |
| PsaM | PF07465 | Photosystem I protein M (PsaM) |
| PsaN | PF05479 | Photosystem I reaction centre subunit N (PSAN or PSI-N) |
| PsaX | PF08078 | PsaX family |
| PsbH | PF00737 | Photosystem II 10 kDa phosphoprotein |
| PsbI | PF02532 | Photosystem II reaction centre I protein (PSII 4.8 kDa protein) |
| PsbJ | PF01788 | PsbJ |
| PsbK | PF02533 | Photosystem II 4 kDa reaction centre component |
| PsbL | PF02419 | PsbL protein |
| PsbM | PF05151 | Photosystem II reaction centre M protein (PsbM) |
| PsbN | PF02468 | Photosystem II reaction centre N protein (psbN) |
| PsbP | PF01789 | PsbP |
| PsbQ | PF05757 | Oxygen evolving enhancer protein 3 (PsbQ) |
| PsbR | PF04725 | Photosystem II 10 kDa polypeptide PsbR |
| PsbT | PF01405 | Photosystem II reaction centre T protein |
| PsbU | PF06514 | Photosystem II 12 kDa extrinsic protein (PsbU) |
| PsbW | PF07123 | Photosystem II reaction centre W protein (PsbW) |
| PsbX | PF06596 | Photosystem II reaction centre X protein (PsbX) |
| PsbY | PF06298 | Photosystem II protein Y (PsbY) |
| PSI_8 | PF00796 | Photosystem I reaction centre subunit VIII |
| PSI_PsaE | PF02427 | Photosystem I reaction centre subunit IV / PsaE |
| PSI_PsaF | PF02507 | Photosystem I reaction centre subunit III |
| PSI_PsaH | PF03244 | Photosystem I reaction centre subunit VI |
| PSI_PsaJ | PF01701 | Photosystem I reaction centre subunit IX / PsaJ |
| PSI_PSAK | PF01241 | Photosystem I psaG / psaK |
| PSII | PF00421 | Photosystem II protein |
| PufQ | PF05398 | PufQ cytochrome subunit |
| RC-P840_PscD | PF10657 | Photosystem P840 reaction centre protein PscD |
| SCO1-SenC | PF02630 | SCO1/SenC |
| TspO_MBR | PF03073 | TspO/MBR family |
| VDE | PF07137 | Violaxanthin de-epoxidase (VDE) |
| Ycf4 | PF02392 | Ycf4 |
