# Supplementary material for: Ecogenomic Perspectives on Domains of Unknown Function: Correlation-Based Exploration of Marine Metagenomes
Source: PLoS One. 2013 Mar 14;8(3):e50869. doi: 10.1371/journal.pone.0050869 (PMC3597751; doi:10.1371/journal.pone.0050869)
Supplement: Table S2 — DUFs with correlative bias towards photobiologically relevant domains (unstandardized data). (DOC) [file pone.0050869.s003.doc]

**Table S2: DUFs with correlative bias towards photobiologically relevant domains (unstandardized data)**

| **DUF** | **Secondary category (fraction of primary category)** |
| --- | --- |
| DUF2808 | Carb (0.22) |
| DUF3386 | Carb, CellDiv, CoE (0.13) |
| DUF3288 | Carb, CellDiv, CoE, E (0.17) |
| DUF1824 | CellDiv (0.13) |
| DUF3120 | CellDiv (0.13) |
| DUF3122 | CellDiv (0.13) |
| DUF3529 | CellDiv (0.13) |
| DUF1818 | CellDiv (0.14) |
| DUF2930 | CellDiv (0.14) |
| DUF3067 | CellDiv (0.14) |
| DUF3086 | CellDiv (0.14) |
| DUF3318 | CellDiv (0.14) |
| DUF1957 | CellDiv (0.17) |
| DUF1997 | CellDiv (0.17) |
| DUF3464 | CellDiv (0.17) |
| DUF3685 | CellDiv (0.17) |
| DUF3146 | CellDiv (0.20) |
| DUF3155 | CellDiv (0.20) |
| DUF2996 | CellDiv (0.25) |
| DUF3119 | CellDiv (0.25) |
| DUF3611 | CellDiv (0.25) |
| DUF2214 | CellDiv (0.5) |
| DUF1350 | CellDiv, CoE (0.13) |
| DUF1400 | CellDiv, CoE (0.13) |
| DUF2854 | CellDiv, CoE (0.13) |
| DUF3172 | CellDiv, CoE (0.13) |
| DUF1651 | CellDiv, CoE (0.14) |
| DUF3082 | CellDiv, CoE (0.14) |
| DUF3153 | CellDiv, CoE (0.14) |
| DUF3177 | CellDiv, CoE (0.14) |
| DUF1823 | CellDiv, CoE (0.33) |
| DUF3181 | CellDiv, CoE, E (0.17) |
| DUF3531 | CellDiv, CoE, E (0.17) |
| DUF2518 | CoE (0.17) |
| DUF697 | CoE (0.22) |
| DUF1092 | CoE (0.25) |
| DUF3353 | CoE (0.25) |
| DUF3007 | CoE (0.29) |
| DUF3066 | CoE (0.33) |
| DUF3769 | CoE (0.33) |
| DUF561 | CoE (0.33) |
| DUF3104 | CoE (0.40) |
| DUF1230 | None |
| DUF1825 | None |
| DUF1995 | None |
| DUF2103 | None |
| DUF2499 | None |
| DUF2839 | None |
| DUF3038 | None |
| DUF3110 | None |
| DUF3303 | None |
| DUF3326 | None |
| DUF3539 | None |
| DUF3571 | None |
| DUF3727 | None |
| DUF98 | None |
