# Supplementary material for: Ecogenomic Perspectives on Domains of Unknown Function: Correlation-Based Exploration of Marine Metagenomes
Source: PLoS One. 2013 Mar 14;8(3):e50869. doi: 10.1371/journal.pone.0050869 (PMC3597751; doi:10.1371/journal.pone.0050869)
Supplement: Table S3 — DUFs with correlative bias towards metabolic categories (standardized data). Refer to Table 1 , footnote for list of abbreviations. (DOC) [file pone.0050869.s004.doc]

**Table S3: DUFs with correlative bias towards metabolic categories (standardized data)**

| **DUF** | **Primary category** | **Secondary category (fraction of primary category)** |
| --- | --- | --- |
| DUF212 | Photo | AA (0.33) |
| DUF3066 |  | AA, CoE (0.20) |
| DUF3479 |  | AA, CoE (0.33) |
| DUF92 |  | AA, CoE, Ion (0.25) |
| DUF3122 |  | Carb (0.33) |
| DUF2499 |  | Carb (0.40) |
| DUF3153 |  | Carb (0.50) |
| DUF3120 |  | Carb, CoE (0.25) |
| DUF3685 |  | Carb, CoE (0.25) |
| DUF2518 |  | Carb, CoE (0.33) |
| DUF3086 |  | Carb, CoE (0.33) |
| DUF3146 |  | Carb, CoE (0.33) |
| DUF2808 |  | Carb, CoE (0.38) |
| DUF1957 |  | Carb, CoE (0.40) |
| DUF1400 |  | Carb, CoE (0.43) |
| DUF3386 |  | Carb, CoE (0.43) |
| DUF3172 |  | Carb, CoE (0.50) |
| DUF1230 |  | Carb, CoE (0.50) |
| DUF2996 |  | Carb, CoE (0.50) |
| DUF3464 |  | Carb, CoE (0.50) |
| DUF3119 |  | CellDiv (0.25) |
| DUF3177 |  | CoE (0.29) |
| DUF3529 |  | CoE (0.33) |
| DUF2130 |  | CoE (0.33) |
| DUF3539 |  | CoE (0.33) |
| DUF3727 |  | CoE (0.40) |
| DUF3353 |  | CoE (0.43) |
| DUF3007 |  | CoE (0.43) |
| DUF1350 |  | CoE (0.50) |
| DUF3571 |  | CoE (0.50) |
| DUF3038 |  | CoE (0.50) |
| DUF1824 |  | CoE (0.50) |
| DUF2930 |  | CoE, Ion (0.25) |
| DUF1823 |  | None |
| DUF1825 |  | None |
| DUF1995 |  | None |
| DUF2358 |  | None |
| DUF2839 |  | None |
| DUF3110 |  | None |
| DUF3155 |  | None |
| DUF3318 |  | None |
| DUF3531 |  | None |
| DUF3593 |  | None |
| DUF512 |  | None |
| DUF98 |  | None |
| DUF111 | Carb | None |
| DUF1008 | Ion | None |
| DUF37 | Transcr | None |
