# Supplementary material for: Ecogenomic Perspectives on Domains of Unknown Function: Correlation-Based Exploration of Marine Metagenomes
Source: PLoS One. 2013 Mar 14;8(3):e50869. doi: 10.1371/journal.pone.0050869 (PMC3597751; doi:10.1371/journal.pone.0050869)
Supplement: Table S4 — Domains present in a network region characterized by photobiologically relevant domains (unstandardized data; Figure 1 , Box 2). Refer to Table 1 , footnote for list of abbreviations. (DOC) [file pone.0050869.s005.doc]

Table S4: Domains present in a network region characterized by photobiologically relevant domains (unstandardized data; Figure 1, Box 2)

| **Category** | **Pfam ID** | **Pfam Comment** |
| --- | --- | --- |
| CellDiv | MinC_C | In Escherichia coli Swiss:P06138 assembles into a Z ring at midcell while assembly at polar sites is prevented by the min system. MinC Swiss:P18196 a component of this system, is an inhibitor of FtsZ assembly that is positioned within the cell by interaction with MinDE. MinC is an oligomer, probably a dimer. The C terminal half of MinC is the most conserved and interacts with MinD. The N terminal half is thought interact with FtsZ. |
| CoE | 2-ph_phosp | Thought to catalyse 2-phosphosulpholactate = sulpholactate + phosphate. Probable magnesium cofactor. Involved in the second step of coenzyme M biosynthesis. Inhibited by vanadate in Methanococcus jannaschii. Also known as the ComB family. |
| NA | DUF1092 | This family consists of several hypothetical proteins of unknown function all from photosynthetic organisms including plants and cyanobacteria. |
|  | DUF1230 | This family consists of several hypothetical plant and photosynthetic bacterial proteins of around 160 residues in length. The function of this family is unknown although looking at the species distribution the protein may play a part in photosynthesis. |
|  | DUF1350 | This family consists of several hypothetical proteins from both cyanobacteria and plants. Members of this family are typically around 250 residues in length. The function of this family is unknown but the species distribution indicates that the family may be involved in photosynthesis. |
|  | DUF1400 | This family contains a number of hypothetical proteins of unknown function that seem to be specific to cyanobacteria. Members of this family have an alpha/beta hydrolase fold. |
|  | DUF1651 | This is a family containing bacterial proteins of unknown function. |
|  | DUF1818 | This presumed domain is found in a small family of cyanobacterial protein. These proteins are functionally uncharacterised. |
|  | DUF1823 | This presumed domain is functionally uncharacterised. |
|  | DUF1824 | This uncharacterised family of proteins are principally found in cyanobacteria. |
|  | DUF1957 | This domain is found in a set of hypothetical bacterial proteins. Its exact function has not, as yet, been defined. |
|  | DUF1995 | This family of proteins are functionally uncharacterised. |
|  | DUF1997 | This family of proteins are functionally uncharacterised. |
|  | DUF2010 | This is a family of proteins which show sequence similarity to the HAD superfamily of hydrolases. |
|  | DUF2103 | This domain, found in various putative metal binding prokaryotic proteins, has no known function. |
|  | DUF2214 | This domain, found in various hypothetical bacterial proteins, has no known function. |
|  | DUF2499 | Members of this family are found in plants, lower eukaryotes, and bacteria and the chloroplast where it is annotated as Ycf49 or Ycf49-like. The function is not known though several members are annotated as putative membrane proteins. |
|  | DUF2518 | This family is conserved in Cyanobacteria. Several members are annotated as the protein Ycf51. The function is not known. |
|  | DUF2808 | This family of proteins with unknown function appears to be restricted to Cyanobacteria. |
|  | DUF2839 | This bacterial family of unknown function appear to be restricted to Cyanobacteria. |
|  | DUF2854 | This family of proteins has no known function. |
|  | DUF2930 | This family of proteins has no known function. |
|  | DUF2996 | This family of proteins has no known function. |
|  | DUF3007 | This is a family of uncharacterised proteins found in bacteria and eukaryotes. |
|  | DUF3038 | This family of proteins with unknown function appear to be restricted to Cyanobacteria. |
|  | DUF3066 | This family of proteins with unknown function appears to be restricted to Cyanobacteria. |
|  | DUF3067 | This family of proteins has no known function. |
|  | DUF3082 | This family of proteins has no known function. |
|  | DUF3086 | This family of proteins with unknown function appears to be restricted to Cyanobacteria. |
|  | DUF3104 | This family of proteins with unknown function appears to be restricted to Cyanobacteria. |
|  | DUF3119 | This family of proteins has no known function. |
|  | DUF3120 | This family of proteins with unknown function appears to be restricted to Cyanobacteria. |
|  | DUF3122 | This family of proteins with unknown function appear to be restricted to Cyanobacteria. |
|  | DUF3146 | This family of proteins with unknown function appear to be restricted to Cyanobacteria. |
|  | DUF3153 | This family of proteins with unknown function appear to be restricted to Cyanobacteria. Some members are annotated as membrane proteins however this cannot be confirmed. |
|  | DUF3155 | This family of proteins with unknown function appears to be restricted to Cyanobacteria. |
|  | DUF3172 | This family of proteins has no known function. |
|  | DUF3177 | Some members in this family of proteins are annotated as membrane proteins however this cannot be confirmed. Currently there is no known function. |
|  | DUF3181 | This family of proteins has no known function. |
|  | DUF3288 | This family of proteins with unknown function appears to be restricted to Cyanobacteria. |
|  | DUF3303 | Several members are annotated as being LysM domain-like proteins, but these did not match any LysM domains reported in the literature. |
|  | DUF3318 | This is a bacterial family of uncharacterised proteins. |
|  | DUF3326 | This protein is functionally uncharacterized. It is about 300-500 amino acids in length. This family is found in plants and bacteria. |
|  | DUF3353 | This family of proteins are functionally uncharacterised. This protein is found in bacteria and eukaryotes. Proteins in this family are typically between 205 to 258 amino acids in length. |
|  | DUF3386 | This family of proteins are functionally uncharacterised. This protein is found in bacteria and eukaryotes. Proteins in this family are about 220 amino acids in length. |
|  | DUF3464 | This family of proteins are functionally uncharacterised. This protein is found in bacteria and eukaryotes. Proteins in this family are typically between 137 to 196 amino acids in length. |
|  | DUF3479 | This presumed domain is functionally uncharacterised. This domain is found in bacteria, archaea and eukaryotes. This domain is about 160 amino acids in length. This domain is found associated with Pfam:PF02514. |
|  | DUF3529 | This family of proteins is functionally uncharacterised. This protein is found in bacteria and eukaryotes. Proteins in this family are typically between 176 to 190 amino acids in length. |
|  | DUF3531 | This family of proteins is functionally uncharacterised. This protein is found in bacteria and eukaryotes. Proteins in this family are typically between 149 to 199 amino acids in length. |
|  | DUF3539 | This family of proteins is functionally uncharacterised. This protein is found in bacteria. Proteins in this family are about 90 amino acids in length. This protein has a conserved NHP sequence motif. |
|  | DUF3571 | This family of proteins is functionally uncharacterised. This protein is found in bacteria and eukaryotes. Proteins in this family are typically between 85 to 97 amino acids in length. |
|  | DUF3593 | This family of proteins is functionally uncharacterised.This family of proteins is found in bacteria and eukaryotes. Proteins in this family are typically between 98 and 228 amino acids in length. There is a conserved LHG sequence motif. |
|  | DUF3611 | This family of proteins is found in bacteria and eukaryotes. Proteins in this family are typically between 180 and 205 amino acids in length. There are two completely conserved residues (W and G) that may be functionally important. |
|  | DUF3641 | This domain family is found in bacteria and eukaryotes, and is approximately 140 amino acids in length. The family is found in association with Pfam:PF04055. This family consists of proteins which are commonly annotated as Radical SAM domains but there is little annotation to back this up. |
|  | DUF3685 | This domain family is found in bacteria and eukaryotes, and is approximately 190 amino acids in length. There are two completely conserved residues (L and D) that may be functionally important. |
|  | DUF3727 | This domain family is found in bacteria and eukaryotes, and is approximately 100 amino acids in length. |
|  | DUF3769 | This family of proteins is found in bacteria and eukaryotes. Proteins in this family are typically between 560 and 931 amino acids in length. |
|  | DUF561 | Protein of unknown function found in a cyanobacterium, and the chloroplasts of algae. |
|  | DUF697 | Family of bacterial hypothetical proteins that is sometimes associated with GTPase domains. |
|  | DUF98 | This is a family of uncharacterised proteins. |
| Photo | Fe_bilin_red | This family consists of several different but closely related proteins which include phycocyanobilin:ferredoxin oxidoreductase EC:1.3.7.5 (PcyA), 15,16-dihydrobiliverdin:ferredoxin oxidoreductase EC:1.3.7.2 (PebA) and phycoerythrobilin:ferredoxin oxidoreductase EC:1.3.7.3 (PebB). Phytobilins are linear tetrapyrrole precursors of the light-harvesting prosthetic groups of the phytochrome photoreceptors of plants and the phycobiliprotein photosynthetic antennae of cyanobacteria, red algae, and cryptomonads. It is known that that phytobilins are synthesised from heme via the intermediary of biliverdin IX alpha (BV), which is reduced subsequently by ferredoxin-dependent bilin reductases with different double-bond specificities. |
|  | MSP | This family consists of the 33 KDa photosystem II polypeptide from the oxygen evolving complex (OEC) of plants and cyanobacteria. The protein is also known as the manganese-stabilising protein as it is associated with the manganese complex of the OEC and may provide the ligands for the complex. |
|  | NdhL | The NdhL family is a component of the NDH-1L complex that is one of the proton-pumping NADH:ubiquinone oxidoreductases that catalyse the electron transfer from NADH to ubiquinone linked with proton translocation across the membrane. NDH-1L is essential for photoheterotrophic cell growth. NdhL appears to contain two transmembrane helices and it is necessary for the functioning of though not the correct assembly of the NDH-1 complex in Synechocystis 6803. The conservation between cyanobacteria and green plants suggests that chloroplast NDH-1 complexes contain related subunits. |
|  | PsaA_PsaB | <NULL> |
|  | PsaD | This family consists of PsaD from plants and cyanobacteria. PsaD is an extrinsic polypeptide of photosystem I (PSI) and is required for native assembly of PSI reaction clusters and is implicated in the electrostatic binding of ferredoxin within the reaction centre. PsaD forms a dimer in solution which is bound by PsaE however PsaD is monomeric in its native complexed PSI environment. |
|  | PsaL | This family consists of the photosystem I reaction centre subunit XI, PsaL, from plants and bacteria. PsaL is one of the smaller subunits in photosystem I with only two transmembrane alpha helices and interacts closely with PsaI. |
|  | PsbP | This family consists of the 23 kDa subunit of oxygen evolving system of photosystem II or PsbP from various plants (where it is encoded by the nuclear genome) and Cyanobacteria. The 23 KDa PsbP protein is required for PSII to be fully operational in vivo, it increases the affinity of the water oxidation site for Cl- and provides the conditions required for high affinity binding of Ca2+. |
|  | PSI_PsaF | Photosystem I (PSI) is an integral membrane protein complex that uses light energy to mediate electron transfer from plastocyanin to ferredoxin. Subunit III (or PSI-F) is one of at least 14 different subunits that compose the PSI complex. |
|  | PSII | <NULL> |
|  | Ycf4 | This family consists of hypothetical Ycf4 proteins from various chloroplast genomes. It has been suggested that Ycf4 is involved in the assembly and/or stability of the photosystem I complex in chloroplasts. |
