# Supplementary material for: Ecogenomic Perspectives on Domains of Unknown Function: Correlation-Based Exploration of Marine Metagenomes
Source: PLoS One. 2013 Mar 14;8(3):e50869. doi: 10.1371/journal.pone.0050869 (PMC3597751; doi:10.1371/journal.pone.0050869)
Supplement: Table S5 — Domains present in a network region bridging two enmeshed regions (unstandardized data; Figure 1 , Box 4). Refer to Table 1 , footnote for list of abbreviations. (DOC) [file pone.0050869.s006.doc]

Table S5: Domains present in a network region bridging two enmeshed regions (unstandardized data; Figure 1, Box 4)

| **Category** | **Pfam ID** | **Pfam Comment** |
| --- | --- | --- |
| Carb | Fructosamin_kin | This family includes eukaryotic fructosamine-3-kinase enzymes. The family also includes bacterial members that have not been characterised but probably have a similar or identical function. |
|  | Phosphorylase | The members of this family catalyse the formation of glucose 1-phosphate from one of the following polyglucoses; glycogen, starch, glucan or maltodextrin. |
|  | RuBisCO_large | The C-terminal domain of RuBisCO large chain is the catalytic domain adopting a TIM barrel fold. |
|  | RuBisCO_large_N | The N-terminal domain of RuBisCO large chain adopts a ferredoxin-like fold. |
| CoE | CbiC | This is a family Precorrin-8X methylmutases also known as Precorrin isomerase, CbiC/CobH, EC:5.4.1.2. This enzyme catalyses the reaction: Precorrin-8X <=> hydrogenobyrinate. This enzyme is part of the Cobalamin (vitamin B12) biosynthetic pathway and catalyses a methyl rearrangement. |
|  | CbiG_C | Members of this family are involved in cobalamin synthesis. The gene encoded by Swiss:P72862 has been designated cbiH but in fact represents a fusion between cbiH and cbiG. As other multi-functional proteins involved in cobalamin biosynthesis catalyse adjacent steps in the pathway, including CysG, CobL (CbiET), CobIJ and CobA-HemD, it is therefore possible that CbiG catalyses a reaction step adjacent to CbiH. In the anaerobic pathway such a step could be the formation of a gamma lactone, which is thought to help to mediate the anaerobic ring contraction process. Within the cobalamin synthesis pathway CbiG catalyses the both the opening of the lactone ring and the extrusion of the two-carbon fragment of cobalt-precorrin-5A from C-20 and its associated methyl group (deacylation) to give cobalt-precorrin-5B. This family is the C-terminal region, and the mid- and N-termival parts are conserved independently in other families. |
|  | CbiJ | This family consists of Precorrin-6x reductase EC:1.3.1.54. This enzyme catalyses the reaction: precorrin-6Y + NADP(+) <=> precorrin-6X + NADPH. CbiJ and CobK both catalyse the reduction of macocycle in the colbalmin biosynthesis pathway. |
|  | CobA_CobO_BtuR | This family consists of the BtuR, CobO, CobP proteins all of which are Cob(I)alamin adenosyltransferase, EC:2.5.1.17, involved in cobalamin (vitamin B12) biosynthesis. These enzymes catalyse the adenosylation reaction: ATP + cob(I)alamin + H2O <=> phosphate + diphosphate + adenosylcobalamin. |
|  | CobD_Cbib | This family includes CobD proteins from a number of bacteria, in Salmonella this protein is called Cbib. Salmonella CobD is a different protein. This protein is involved in cobalamin biosynthesis and is probably an enzyme responsible for the conversion of adenosylcobyric acid to adenosylcobinamide or adenosylcobinamide phosphate. |
|  | CobN-Mg_chel | This family contains a domain common to the cobN protein and to magnesium protoporphyrin chelatase. CobN is implicated in the conversion of hydrogenobyrinic acid a,c-diamide to cobyrinic acid. Magnesium protoporphyrin chelatase is involved in chlorophyll biosynthesis. |
|  | CobS | This is family of Colbalmin-5-phosphate synthases, CobS, from bacteria. The CobS enzyme catalyses the synthesis of AdoCbl-5'-p from AdoCbi-GDP and alpha-ribazole-5'-P. This enzyme is involved in the cobalamin (vitamin B12) biosynthesis pathway in particular the nucleotide loop assembly stage in conjunction with CobC, CobU and CobT. |
| E | RuBisCO_small | <NULL> |
| NA | DUF1499 | This family consists of several hypothetical bacterial and plant proteins of around 125 residues in length. The function of this family is unknown. |
|  | DUF212 | This family is related to the Pfam:PF01569 family (personal obs: C Yeats). |
|  | DUF2130 | This domain, found in various hypothetical prokaryotic proteins, has no known function. |
|  | DUF512 | Family of uncharacterised prokaryotic proteins. |
