# Supplementary material for: Ecogenomic Perspectives on Domains of Unknown Function: Correlation-Based Exploration of Marine Metagenomes
Source: PLoS One. 2013 Mar 14;8(3):e50869. doi: 10.1371/journal.pone.0050869 (PMC3597751; doi:10.1371/journal.pone.0050869)
Supplement: Table S6 — Domains present in the primary network generated from standardized Pfam abundances across GOS sites ( Figure 2, i ). Refer to Table 1 , footnote for list of abbreviations. (DOC) [file pone.0050869.s007.doc]

Table S6: Domains present in the primary network generated from standardized Pfam abundances across GOS sites (Figure 2, i)

| **Category** | **Pfam ID** | **Pfam Comment** |
| --- | --- | --- |
| AA | Bact_transglu_N | This region is found towards the N-terminus of various archaeal and bacterial hypothetical proteins. Some of these are annotated as being transglutaminase-like proteins, and in fact contain a transglutaminase-like superfamily domain (Pfam:PF01841). |
|  | Peptidase_M32 | <NULL> |
|  | Urease_alpha | The N-terminal domain is a composite domain and plays a major trimer stabilising role by contacting the catalytic domain of the symmetry related alpha-subunit. |
|  | Urease_beta | This subunit is known as alpha in Heliobacter. |
| Carb | Alpha-amylase | Alpha amylase is classified as family 13 of the glycosyl hydrolases. The structure is an 8 stranded alpha/beta barrel containing the active site, interrupted by a ~70 a.a. calcium-binding domain protruding between beta strand 3 and alpha helix 3, and a carboxyl-terminal Greek key beta-barrel domain. |
|  | Fructosamin_kin | This family includes eukaryotic fructosamine-3-kinase enzymes. The family also includes bacterial members that have not been characterised but probably have a similar or identical function. |
|  | Glyco_hydro_77 | These enzymes EC:2.4.1.25 transfer a segment of a (1,4)-alpha-D-glucan to a new 4-position in an acceptor, which may be glucose or (1,4)-alpha-D-glucan. |
|  | Phosphorylase | The members of this family catalyse the formation of glucose 1-phosphate from one of the following polyglucoses; glycogen, starch, glucan or maltodextrin. |
|  | RuBisCO_large | The C-terminal domain of RuBisCO large chain is the catalytic domain adopting a TIM barrel fold. |
|  | RuBisCO_large_N | The N-terminal domain of RuBisCO large chain adopts a ferredoxin-like fold. |
| CellDiv | MinC_C | In Escherichia coli Swiss:P06138 assembles into a Z ring at midcell while assembly at polar sites is prevented by the min system. MinC Swiss:P18196 a component of this system, is an inhibitor of FtsZ assembly that is positioned within the cell by interaction with MinDE. MinC is an oligomer, probably a dimer. The C terminal half of MinC is the most conserved and interacts with MinD. The N terminal half is thought interact with FtsZ. |
| CoE | 2-ph_phosp | Thought to catalyse 2-phosphosulpholactate = sulpholactate + phosphate. Probable magnesium cofactor. Involved in the second step of coenzyme M biosynthesis. Inhibited by vanadate in Methanococcus jannaschii. Also known as the ComB family. |
|  | CbiD | CbiD is essential for cobalamin biosynthesis in both S. typhimurium and B. megaterium, no functional role has been ascribed to the protein. The CbiD protein has a putative S-AdoMet binding site. It is possible that CbiD might have the same role as CobF in undertaking the C-1 methylation and deacylation reactions required during the ring contraction process. |
|  | CbiG_C | Members of this family are involved in cobalamin synthesis. The gene encoded by Swiss:P72862 has been designated cbiH but in fact represents a fusion between cbiH and cbiG. As other multi-functional proteins involved in cobalamin biosynthesis catalyse adjacent steps in the pathway, including CysG, CobL (CbiET), CobIJ and CobA-HemD, it is therefore possible that CbiG catalyses a reaction step adjacent to CbiH. In the anaerobic pathway such a step could be the formation of a gamma lactone, which is thought to help to mediate the anaerobic ring contraction process. Within the cobalamin synthesis pathway CbiG catalyses the both the opening of the lactone ring and the extrusion of the two-carbon fragment of cobalt-precorrin-5A from C-20 and its associated methyl group (deacylation) to give cobalt-precorrin-5B. This family is the C-terminal region, and the mid- and N-termival parts are conserved independently in other families. |
|  | CbiJ | This family consists of Precorrin-6x reductase EC:1.3.1.54. This enzyme catalyses the reaction: precorrin-6Y + NADP(+) <=> precorrin-6X + NADPH. CbiJ and CobK both catalyse the reduction of macocycle in the colbalmin biosynthesis pathway. |
|  | CbiK | This family consists of several bacterial cobalt chelatase (CbiK) proteins (EC:4.99.1.-). |
|  | CobA_CobO_BtuR | This family consists of the BtuR, CobO, CobP proteins all of which are Cob(I)alamin adenosyltransferase, EC:2.5.1.17, involved in cobalamin (vitamin B12) biosynthesis. These enzymes catalyse the adenosylation reaction: ATP + cob(I)alamin + H2O <=> phosphate + diphosphate + adenosylcobalamin. |
|  | CobN-Mg_chel | This family contains a domain common to the cobN protein and to magnesium protoporphyrin chelatase. CobN is implicated in the conversion of hydrogenobyrinic acid a,c-diamide to cobyrinic acid. Magnesium protoporphyrin chelatase is involved in chlorophyll biosynthesis. |
|  | CobS | This is family of Colbalmin-5-phosphate synthases, CobS, from bacteria. The CobS enzyme catalyses the synthesis of AdoCbl-5'-p from AdoCbi-GDP and alpha-ribazole-5'-P. This enzyme is involved in the cobalamin (vitamin B12) biosynthesis pathway in particular the nucleotide loop assembly stage in conjunction with CobC, CobU and CobT. |
|  | DBI_PRT | This family of proteins represent the nicotinate-nucleotide- dimethylbenzimidazole phosphoribosyltransferase (NN:DBI PRT) enzymes involved in dimethylbenzimidazole synthesis. This function is essential to de novo cobalamin (vitamin B12) production in bacteria. Nicotinate mononucleotide (NaMN):5,6-dimethylbenzimidazole (DMB) phosphoribosyltransferase (CobT) from Salmonella enterica plays a central role in the synthesis of alpha-ribazole-5'-phosphate, an intermediate for the lower ligand of cobalamin. |
| E | Oxidored_nitro | <NULL> |
|  | Pyrophosphatase | <NULL> |
|  | RuBisCO_small | <NULL> |
| Ion | CutA1 | Several gene loci with a possible involvement in cellular tolerance to copper have been identified. One such locus in eubacteria and archaebacteria, cutA, is thought to be involved in cellular tolerance to a wide variety of divalent cations other than copper. The cutA locus consists of two operons, of one and two genes. The CutA1 protein is a cytoplasmic protein, encoded by the single-gene operon and has been linked to divalent cation tolerance. It has no recognised structural motifs. This family also contains putative proteins from eukaryotes (human and Drosophila). |
|  | Voltage_CLC | This family of ion channels contains 10 or 12 transmembrane helices. Each protein forms a single pore. It has been shown that some members of this family form homodimers. In terms of primary structure, they are unrelated to known cation channels or other types of anion channels. Three ClC subfamilies are found in animals. ClC-1 (Swiss:P35523) is involved in setting and restoring the resting membrane potential of skeletal muscle, while other channels play important parts in solute concentration mechanisms in the kidney. These proteins contain two Pfam:PF00571 domains. |
| NA | DUF1092 | This family consists of several hypothetical proteins of unknown function all from photosynthetic organisms including plants and cyanobacteria. |
|  | DUF111 | This prokaryotic family has no known function. |
|  | DUF1230 | This family consists of several hypothetical plant and photosynthetic bacterial proteins of around 160 residues in length. The function of this family is unknown although looking at the species distribution the protein may play a part in photosynthesis. |
|  | DUF1257 | This family contains hypothetical proteins of unknown function that are approximately 120 residues long. Family members include eukaryotic and bacterial proteins. |
|  | DUF1350 | This family consists of several hypothetical proteins from both cyanobacteria and plants. Members of this family are typically around 250 residues in length. The function of this family is unknown but the species distribution indicates that the family may be involved in photosynthesis. |
|  | DUF1400 | This family contains a number of hypothetical proteins of unknown function that seem to be specific to cyanobacteria. Members of this family have an alpha/beta hydrolase fold. |
|  | DUF1499 | This family consists of several hypothetical bacterial and plant proteins of around 125 residues in length. The function of this family is unknown. |
|  | DUF1651 | This is a family containing bacterial proteins of unknown function. |
|  | DUF1818 | This presumed domain is found in a small family of cyanobacterial protein. These proteins are functionally uncharacterised. |
|  | DUF1823 | This presumed domain is functionally uncharacterised. |
|  | DUF1824 | This uncharacterised family of proteins are principally found in cyanobacteria. |
|  | DUF1957 | This domain is found in a set of hypothetical bacterial proteins. Its exact function has not, as yet, been defined. |
|  | DUF1995 | This family of proteins are functionally uncharacterised. |
|  | DUF1997 | This family of proteins are functionally uncharacterised. |
|  | DUF2010 | This is a family of proteins which show sequence similarity to the HAD superfamily of hydrolases. |
|  | DUF212 | This family is related to the Pfam:PF01569 family (personal obs: C Yeats). |
|  | DUF2130 | This domain, found in various hypothetical prokaryotic proteins, has no known function. |
|  | DUF2214 | This domain, found in various hypothetical bacterial proteins, has no known function. |
|  | DUF2358 | DUF2358 is a family of conserved proteins found from plants to humans. The function is unknown. |
|  | DUF2499 | Members of this family are found in plants, lower eukaryotes, and bacteria and the chloroplast where it is annotated as Ycf49 or Ycf49-like. The function is not known though several members are annotated as putative membrane proteins. |
|  | DUF2518 | This family is conserved in Cyanobacteria. Several members are annotated as the protein Ycf51. The function is not known. |
|  | DUF2808 | This family of proteins with unknown function appears to be restricted to Cyanobacteria. |
|  | DUF2834 | This is a bacterial family of uncharacterised proteins. |
|  | DUF2839 | This bacterial family of unknown function appear to be restricted to Cyanobacteria. |
|  | DUF2854 | This family of proteins has no known function. |
|  | DUF2930 | This family of proteins has no known function. |
|  | DUF2996 | This family of proteins has no known function. |
|  | DUF3007 | This is a family of uncharacterised proteins found in bacteria and eukaryotes. |
|  | DUF3038 | This family of proteins with unknown function appear to be restricted to Cyanobacteria. |
|  | DUF3066 | This family of proteins with unknown function appears to be restricted to Cyanobacteria. |
|  | DUF3067 | This family of proteins has no known function. |
|  | DUF3082 | This family of proteins has no known function. |
|  | DUF3086 | This family of proteins with unknown function appears to be restricted to Cyanobacteria. |
|  | DUF3104 | This family of proteins with unknown function appears to be restricted to Cyanobacteria. |
|  | DUF3119 | This family of proteins has no known function. |
|  | DUF3120 | This family of proteins with unknown function appears to be restricted to Cyanobacteria. |
|  | DUF3122 | This family of proteins with unknown function appear to be restricted to Cyanobacteria. |
|  | DUF3146 | This family of proteins with unknown function appear to be restricted to Cyanobacteria. |
|  | DUF3153 | This family of proteins with unknown function appear to be restricted to Cyanobacteria. Some members are annotated as membrane proteins however this cannot be confirmed. |
|  | DUF3155 | This family of proteins with unknown function appears to be restricted to Cyanobacteria. |
|  | DUF3172 | This family of proteins has no known function. |
|  | DUF3177 | Some members in this family of proteins are annotated as membrane proteins however this cannot be confirmed. Currently there is no known function. |
|  | DUF3181 | This family of proteins has no known function. |
|  | DUF3288 | This family of proteins with unknown function appears to be restricted to Cyanobacteria. |
|  | DUF3303 | Several members are annotated as being LysM domain-like proteins, but these did not match any LysM domains reported in the literature. |
|  | DUF3318 | This is a bacterial family of uncharacterised proteins. |
|  | DUF3326 | This protein is functionally uncharacterized. It is about 300-500 amino acids in length. This family is found in plants and bacteria. |
|  | DUF3353 | This family of proteins are functionally uncharacterised. This protein is found in bacteria and eukaryotes. Proteins in this family are typically between 205 to 258 amino acids in length. |
|  | DUF3386 | This family of proteins are functionally uncharacterised. This protein is found in bacteria and eukaryotes. Proteins in this family are about 220 amino acids in length. |
|  | DUF3464 | This family of proteins are functionally uncharacterised. This protein is found in bacteria and eukaryotes. Proteins in this family are typically between 137 to 196 amino acids in length. |
|  | DUF3479 | This presumed domain is functionally uncharacterised. This domain is found in bacteria, archaea and eukaryotes. This domain is about 160 amino acids in length. This domain is found associated with Pfam:PF02514. |
|  | DUF3529 | This family of proteins is functionally uncharacterised. This protein is found in bacteria and eukaryotes. Proteins in this family are typically between 176 to 190 amino acids in length. |
|  | DUF3531 | This family of proteins is functionally uncharacterised. This protein is found in bacteria and eukaryotes. Proteins in this family are typically between 149 to 199 amino acids in length. |
|  | DUF3539 | This family of proteins is functionally uncharacterised. This protein is found in bacteria. Proteins in this family are about 90 amino acids in length. This protein has a conserved NHP sequence motif. |
|  | DUF3571 | This family of proteins is functionally uncharacterised. This protein is found in bacteria and eukaryotes. Proteins in this family are typically between 85 to 97 amino acids in length. |
|  | DUF3593 | This family of proteins is functionally uncharacterised.This family of proteins is found in bacteria and eukaryotes. Proteins in this family are typically between 98 and 228 amino acids in length. There is a conserved LHG sequence motif. |
|  | DUF3611 | This family of proteins is found in bacteria and eukaryotes. Proteins in this family are typically between 180 and 205 amino acids in length. There are two completely conserved residues (W and G) that may be functionally important. |
|  | DUF3641 | This domain family is found in bacteria and eukaryotes, and is approximately 140 amino acids in length. The family is found in association with Pfam:PF04055. This family consists of proteins which are commonly annotated as Radical SAM domains but there is little annotation to back this up. |
|  | DUF3685 | This domain family is found in bacteria and eukaryotes, and is approximately 190 amino acids in length. There are two completely conserved residues (L and D) that may be functionally important. |
|  | DUF3727 | This domain family is found in bacteria and eukaryotes, and is approximately 100 amino acids in length. |
|  | DUF3769 | This family of proteins is found in bacteria and eukaryotes. Proteins in this family are typically between 560 and 931 amino acids in length. |
|  | DUF403 | <NULL> |
|  | DUF404 | <NULL> |
|  | DUF407 | <NULL> |
|  | DUF490 | <NULL> |
|  | DUF512 | Family of uncharacterised prokaryotic proteins. |
|  | DUF561 | Protein of unknown function found in a cyanobacterium, and the chloroplasts of algae. |
|  | DUF697 | Family of bacterial hypothetical proteins that is sometimes associated with GTPase domains. |
|  | DUF836 | These proteins are related to the Pfam:PF00462 family. |
|  | DUF92 | Members of this family have several predicted transmembrane helices. The function of these prokaryotic proteins is unknown. |
|  | DUF98 | This is a family of uncharacterised proteins. |
| Photo | Fe_bilin_red | This family consists of several different but closely related proteins which include phycocyanobilin:ferredoxin oxidoreductase EC:1.3.7.5 (PcyA), 15,16-dihydrobiliverdin:ferredoxin oxidoreductase EC:1.3.7.2 (PebA) and phycoerythrobilin:ferredoxin oxidoreductase EC:1.3.7.3 (PebB). Phytobilins are linear tetrapyrrole precursors of the light-harvesting prosthetic groups of the phytochrome photoreceptors of plants and the phycobiliprotein photosynthetic antennae of cyanobacteria, red algae, and cryptomonads. It is known that that phytobilins are synthesised from heme via the intermediary of biliverdin IX alpha (BV), which is reduced subsequently by ferredoxin-dependent bilin reductases with different double-bond specificities. |
|  | MSP | This family consists of the 33 KDa photosystem II polypeptide from the oxygen evolving complex (OEC) of plants and cyanobacteria. The protein is also known as the manganese-stabilising protein as it is associated with the manganese complex of the OEC and may provide the ligands for the complex. |
|  | NdhL | The NdhL family is a component of the NDH-1L complex that is one of the proton-pumping NADH:ubiquinone oxidoreductases that catalyse the electron transfer from NADH to ubiquinone linked with proton translocation across the membrane. NDH-1L is essential for photoheterotrophic cell growth. NdhL appears to contain two transmembrane helices and it is necessary for the functioning of though not the correct assembly of the NDH-1 complex in Synechocystis 6803. The conservation between cyanobacteria and green plants suggests that chloroplast NDH-1 complexes contain related subunits. |
|  | PsaA_PsaB | <NULL> |
|  | PsaD | This family consists of PsaD from plants and cyanobacteria. PsaD is an extrinsic polypeptide of photosystem I (PSI) and is required for native assembly of PSI reaction clusters and is implicated in the electrostatic binding of ferredoxin within the reaction centre. PsaD forms a dimer in solution which is bound by PsaE however PsaD is monomeric in its native complexed PSI environment. |
|  | PsaL | This family consists of the photosystem I reaction centre subunit XI, PsaL, from plants and bacteria. PsaL is one of the smaller subunits in photosystem I with only two transmembrane alpha helices and interacts closely with PsaI. |
|  | PsbP | This family consists of the 23 kDa subunit of oxygen evolving system of photosystem II or PsbP from various plants (where it is encoded by the nuclear genome) and Cyanobacteria. The 23 KDa PsbP protein is required for PSII to be fully operational in vivo, it increases the affinity of the water oxidation site for Cl- and provides the conditions required for high affinity binding of Ca2+. |
|  | PSI_PsaF | Photosystem I (PSI) is an integral membrane protein complex that uses light energy to mediate electron transfer from plastocyanin to ferredoxin. Subunit III (or PSI-F) is one of at least 14 different subunits that compose the PSI complex. |
|  | PSII | <NULL> |
|  | Ycf4 | This family consists of hypothetical Ycf4 proteins from various chloroplast genomes. It has been suggested that Ycf4 is involved in the assembly and/or stability of the photosystem I complex in chloroplasts. |
| PostModChaps | ResB | This family includes both ResB and cytochrome c biogenesis proteins. Mutations in ResB indicate that they are essential for growth. ResB is predicted to be a transmembrane protein. |
|  | UreD | UreD is a urease accessory protein. Urease Pfam:PF00449 hydrolyses urea into ammonia and carbamic acid. UreD is involved in activation of the urease enzyme via the UreD-UreF-UreG-urease complex and is required for urease nickel metallocenter assembly. See also UreF Pfam:PF01730, UreG Pfam:PF01495. |
|  | UreF | This family consists of the Urease accessory protein UreF. The urease enzyme (urea amidohydrolase) hydrolyses urea into ammonia and carbamic acid. UreF is proposed to modulate the activation process of urease by eliminating the binding of nickel irons to noncarbamylated protein. |
| RRR | DNA_ligase_A_C | This region is found in many but not all ATP-dependent DNA ligase enzymes (EC:6.5.1.1). It is thought to constitute part of the catalytic core of ATP dependent DNA ligase. |
|  | Exonuc_V_gamma | The Exodeoxyribonuclease V enzyme is a multi-subunit enzyme comprised of the proteins RecB, RecC (this family) and RecD. This enzyme plays an important role in homologous genetic recombination, repair of double strand DNA breaks resistance to UV irradiation and chemical DNA-damage. The enzyme (EC:3.1.11.5) catalyses ssDNA or dsDNA-dependent ATP hydrolysis, hydrolysis of ssDNA or dsDNA and unwinding of dsDNA. This family consists of two AAA domains. |
|  | Pur_DNA_glyco | Methylpurine-DNA glycosylase is a base excision-repair protein. It is responsible for the hydrolysis of the deoxyribose N-glycosidic bond, excising 3-methyladenine and 3-methylguanine from damaged DNA. |
