# Supplementary material for: Ecogenomic Perspectives on Domains of Unknown Function: Correlation-Based Exploration of Marine Metagenomes
Source: PLoS One. 2013 Mar 14;8(3):e50869. doi: 10.1371/journal.pone.0050869 (PMC3597751; doi:10.1371/journal.pone.0050869)
Supplement: Table S7 — Pfam domains contained in the second largest transitivity cluster derived from unstandardized domain abundances ( Figure 3 : TC2). Refer to Table 1 , footnote for list of abbreviations. (DOC) [file pone.0050869.s008.doc]

**Table S7: Pfam domains contained in the second largest transitivity cluster derived from unstandardized domain abundances (Figure 3: TC2)**

| **Category** | **Pfam ID** | **Pfam Comment** |
| --- | --- | --- |
| NA | DUF1092 | This family consists of several hypothetical proteins of unknown function all from photosynthetic organisms including plants and cyanobacteria. |
|  | DUF1230 | This family consists of several hypothetical plant and photosynthetic bacterial proteins of around 160 residues in length. The function of this family is unknown although looking at the species distribution the protein may play a part in photosynthesis. |
|  | DUF1350 | This family consists of several hypothetical proteins from both cyanobacteria and plants. Members of this family are typically around 250 residues in length. The function of this family is unknown but the species distribution indicates that the family may be involved in photosynthesis. |
|  | DUF1400 | This family contains a number of hypothetical proteins of unknown function that seem to be specific to cyanobacteria. Members of this family have an alpha/beta hydrolase fold. |
|  | DUF1651 | This is a family containing bacterial proteins of unknown function. |
|  | DUF1824 | This uncharacterised family of proteins are principally found in cyanobacteria. |
|  | DUF2518 | This family is conserved in Cyanobacteria. Several members are annotated as the protein Ycf51. The function is not known. |
|  | DUF2808 | This family of proteins with unknown function appears to be restricted to Cyanobacteria. |
|  | DUF2854 | This family of proteins has no known function. |
|  | DUF2930 | This family of proteins has no known function. |
|  | DUF3007 | This is a family of uncharacterised proteins found in bacteria and eukaryotes. |
|  | DUF3082 | This family of proteins has no known function. |
|  | DUF3120 | This family of proteins with unknown function appears to be restricted to Cyanobacteria. |
|  | DUF3122 | This family of proteins with unknown function appear to be restricted to Cyanobacteria. |
|  | DUF3153 | This family of proteins with unknown function appear to be restricted to Cyanobacteria. Some members are annotated as membrane proteins however this cannot be confirmed. |
|  | DUF3172 | This family of proteins has no known function. |
|  | DUF3181 | This family of proteins has no known function. |
|  | DUF3288 | This family of proteins with unknown function appears to be restricted to Cyanobacteria. |
|  | DUF3318 | This is a bacterial family of uncharacterised proteins. |
|  | DUF3326 | This protein is functionally uncharacterized. It is about 300-500 amino acids in length. This family is found in plants and bacteria. |
|  | DUF3353 | This family of proteins are functionally uncharacterised. This protein is found in bacteria and eukaryotes. Proteins in this family are typically between 205 to 258 amino acids in length. |
|  | DUF3386 | This family of proteins are functionally uncharacterised. This protein is found in bacteria and eukaryotes. Proteins in this family are about 220 amino acids in length. |
|  | DUF3464 | This family of proteins are functionally uncharacterised. This protein is found in bacteria and eukaryotes. Proteins in this family are typically between 137 to 196 amino acids in length. |
|  | DUF3529 | This family of proteins is functionally uncharacterised. This protein is found in bacteria and eukaryotes. Proteins in this family are typically between 176 to 190 amino acids in length. |
|  | DUF3685 | This domain family is found in bacteria and eukaryotes, and is approximately 190 amino acids in length. There are two completely conserved residues (L and D) that may be functionally important. |
|  | DUF3769 | This family of proteins is found in bacteria and eukaryotes. Proteins in this family are typically between 560 and 931 amino acids in length. |
|  | DUF561 | Protein of unknown function found in a cyanobacterium, and the chloroplasts of algae. |
|  | DUF697 | Family of bacterial hypothetical proteins that is sometimes associated with GTPase domains. |
| Photo | Fe_bilin_red | This family consists of several different but closely related proteins which include phycocyanobilin:ferredoxin oxidoreductase EC:1.3.7.5 (PcyA), 15,16-dihydrobiliverdin:ferredoxin oxidoreductase EC:1.3.7.2 (PebA) and phycoerythrobilin:ferredoxin oxidoreductase EC:1.3.7.3 (PebB). Phytobilins are linear tetrapyrrole precursors of the light-harvesting prosthetic groups of the phytochrome photoreceptors of plants and the phycobiliprotein photosynthetic antennae of cyanobacteria, red algae, and cryptomonads. It is known that that phytobilins are synthesised from heme via the intermediary of biliverdin IX alpha (BV), which is reduced subsequently by ferredoxin-dependent bilin reductases with different double-bond specificities. |
|  | MSP | This family consists of the 33 KDa photosystem II polypeptide from the oxygen evolving complex (OEC) of plants and cyanobacteria. The protein is also known as the manganese-stabilising protein as it is associated with the manganese complex of the OEC and may provide the ligands for the complex. |
|  | PsaA_PsaB | <NULL> |
|  | PSII | <NULL> |
