# Supplementary material for: Ecogenomic Perspectives on Domains of Unknown Function: Correlation-Based Exploration of Marine Metagenomes
Source: PLoS One. 2013 Mar 14;8(3):e50869. doi: 10.1371/journal.pone.0050869 (PMC3597751; doi:10.1371/journal.pone.0050869)
Supplement: Table S8 — Pfam domains contained in the largest transitivity cluster derived from standardized domain abundances ( Figure 4 : TC1). Refer to Table 1 , footnote for list of abbreviations. (DOC) [file pone.0050869.s009.doc]

Table S8: Pfam domains contained in the largest transitivity cluster derived from standardized domain abundances (Figure 4: TC1)

| **Category** | **Pfam ID** | **Pfam Comment** |
| --- | --- | --- |
| Carb | Fructosamin_kin | This family includes eukaryotic fructosamine-3-kinase enzymes. The family also includes bacterial members that have not been characterised but probably have a similar or identical function. |
| CoE | CobA_CobO_BtuR | This family consists of the BtuR, CobO, CobP proteins all of which are Cob(I)alamin adenosyltransferase, EC:2.5.1.17, involved in cobalamin (vitamin B12) biosynthesis. These enzymes catalyse the adenosylation reaction: ATP + cob(I)alamin + H2O <=> phosphate + diphosphate + adenosylcobalamin. |
|  | CobN-Mg_chel | This family contains a domain common to the cobN protein and to magnesium protoporphyrin chelatase. CobN is implicated in the conversion of hydrogenobyrinic acid a,c-diamide to cobyrinic acid. Magnesium protoporphyrin chelatase is involved in chlorophyll biosynthesis. |
| NA | DUF1092 | This family consists of several hypothetical proteins of unknown function all from photosynthetic organisms including plants and cyanobacteria. |
|  | DUF1350 | This family consists of several hypothetical proteins from both cyanobacteria and plants. Members of this family are typically around 250 residues in length. The function of this family is unknown but the species distribution indicates that the family may be involved in photosynthesis. |
|  | DUF1400 | This family contains a number of hypothetical proteins of unknown function that seem to be specific to cyanobacteria. Members of this family have an alpha/beta hydrolase fold. |
|  | DUF1651 | This is a family containing bacterial proteins of unknown function. |
|  | DUF1957 | This domain is found in a set of hypothetical bacterial proteins. Its exact function has not, as yet, been defined. |
|  | DUF1997 | This family of proteins are functionally uncharacterised. |
|  | DUF2518 | This family is conserved in Cyanobacteria. Several members are annotated as the protein Ycf51. The function is not known. |
|  | DUF2808 | This family of proteins with unknown function appears to be restricted to Cyanobacteria. |
|  | DUF2854 | This family of proteins has no known function. |
|  | DUF2996 | This family of proteins has no known function. |
|  | DUF3007 | This is a family of uncharacterised proteins found in bacteria and eukaryotes. |
|  | DUF3038 | This family of proteins with unknown function appear to be restricted to Cyanobacteria. |
|  | DUF3082 | This family of proteins has no known function. |
|  | DUF3086 | This family of proteins with unknown function appears to be restricted to Cyanobacteria. |
|  | DUF3120 | This family of proteins with unknown function appears to be restricted to Cyanobacteria. |
|  | DUF3172 | This family of proteins has no known function. |
|  | DUF3181 | This family of proteins has no known function. |
|  | DUF3288 | This family of proteins with unknown function appears to be restricted to Cyanobacteria. |
|  | DUF3326 | This protein is functionally uncharacterized. It is about 300-500 amino acids in length. This family is found in plants and bacteria. |
|  | DUF3353 | This family of proteins are functionally uncharacterised. This protein is found in bacteria and eukaryotes. Proteins in this family are typically between 205 to 258 amino acids in length. |
|  | DUF3386 | This family of proteins are functionally uncharacterised. This protein is found in bacteria and eukaryotes. Proteins in this family are about 220 amino acids in length. |
|  | DUF3464 | This family of proteins are functionally uncharacterised. This protein is found in bacteria and eukaryotes. Proteins in this family are typically between 137 to 196 amino acids in length. |
|  | DUF3529 | This family of proteins is functionally uncharacterised. This protein is found in bacteria and eukaryotes. Proteins in this family are typically between 176 to 190 amino acids in length. |
|  | DUF3571 | This family of proteins is functionally uncharacterised. This protein is found in bacteria and eukaryotes. Proteins in this family are typically between 85 to 97 amino acids in length. |
|  | DUF3611 | This family of proteins is found in bacteria and eukaryotes. Proteins in this family are typically between 180 and 205 amino acids in length. There are two completely conserved residues (W and G) that may be functionally important. |
|  | DUF3769 | This family of proteins is found in bacteria and eukaryotes. Proteins in this family are typically between 560 and 931 amino acids in length. |
|  | DUF561 | Protein of unknown function found in a cyanobacterium, and the chloroplasts of algae. |
|  | DUF697 | Family of bacterial hypothetical proteins that is sometimes associated with GTPase domains. |
| Photo | Fe_bilin_red | This family consists of several different but closely related proteins which include phycocyanobilin:ferredoxin oxidoreductase EC:1.3.7.5 (PcyA), 15,16-dihydrobiliverdin:ferredoxin oxidoreductase EC:1.3.7.2 (PebA) and phycoerythrobilin:ferredoxin oxidoreductase EC:1.3.7.3 (PebB). Phytobilins are linear tetrapyrrole precursors of the light-harvesting prosthetic groups of the phytochrome photoreceptors of plants and the phycobiliprotein photosynthetic antennae of cyanobacteria, red algae, and cryptomonads. It is known that that phytobilins are synthesised from heme via the intermediary of biliverdin IX alpha (BV), which is reduced subsequently by ferredoxin-dependent bilin reductases with different double-bond specificities. |
|  | MSP | This family consists of the 33 KDa photosystem II polypeptide from the oxygen evolving complex (OEC) of plants and cyanobacteria. The protein is also known as the manganese-stabilising protein as it is associated with the manganese complex of the OEC and may provide the ligands for the complex. |
|  | PsaA_PsaB | <NULL> |
|  | PsbP | This family consists of the 23 kDa subunit of oxygen evolving system of photosystem II or PsbP from various plants (where it is encoded by the nuclear genome) and Cyanobacteria. The 23 KDa PsbP protein is required for PSII to be fully operational in vivo, it increases the affinity of the water oxidation site for Cl- and provides the conditions required for high affinity binding of Ca2+. |
|  | PSII | <NULL> |
