# Supplementary material for: Ecogenomic Perspectives on Domains of Unknown Function: Correlation-Based Exploration of Marine Metagenomes
Source: PLoS One. 2013 Mar 14;8(3):e50869. doi: 10.1371/journal.pone.0050869 (PMC3597751; doi:10.1371/journal.pone.0050869)
Supplement: Table S9 — Pfam domains contained in the selected transitivity clusters derived from unstandardized domain abundances ( Figure 3 ). Refer to Table 1 , footnote for list of abbreviations. (DOC) [file pone.0050869.s010.doc]

Table S9: Pfam domains contained in the selected transitivity clusters derived from unstandardized domain abundances (Figure 3)

| **Cluster** | **Category** | **Pfam ID** | **Pfam Comment** |
| --- | --- | --- | --- |
| TC 7 | NA | DUF37 | This domain is found in short (70 amino acid) hypothetical proteins from various bacteria. The domain contains three conserved cysteine residues. Swiss:Q44066 from Aeromonas hydrophila has been found to have hemolytic activity (unpublished). |
|  | PostModChaps | NifU | This is an alignment of the carboxy-terminal domain. This is the only common region between the NifU protein from nitrogen-fixing bacteria and rhodobacterial species. The biochemical function of NifU is unknown. |
|  | Transcr | RNA_pol_Rpb2_3 | RNA polymerases catalyse the DNA dependent polymerisation of RNA. Prokaryotes contain a single RNA polymerase compared to three in eukaryotes (not including mitochondrial. and chloroplast polymerases). Domain 3, s also known as the fork domain and is proximal to catalytic site. |
|  |  | Sigma70_r2 | Region 2 of sigma-70 is the most conserved region of the entire protein. All members of this class of sigma-factor contain region 2. The high conservation is due to region 2 containing both the -10 promoter recognition helix and the primary core RNA polymerase binding determinant. The core binding helix, interacts with the clamp domain of the largest polymerase subunit, beta prime. The aromatic residues of the recognition helix, found at the C-terminus of this domain are though to mediate strand separation, thereby allowing transcription initiation. |
|  | TransR | Ribosomal_S17 | <NULL> |
|  |  | RNase_PH_C | This family includes 3'-5' exoribonucleases. Ribonuclease PH contains a single copy of this domain, and removes nucleotide residues following the -CCA terminus of tRNA. Polyribonucleotide nucleotidyltransferase (PNPase) contains two tandem copies of the domain. PNPase is involved in mRNA degradation in a 3'-5' direction. The exosome is a 3'-5' exoribonuclease complex that is required for 3' processing of the 5.8S rRNA. Three of its five protein components, Swiss:P46948 Swiss:Q12277 and Swiss:P25359 contain a copy of this domain. Swiss:Q10205, a hypothetical protein from S. pombe appears to belong to an uncharacterised subfamily. This subfamily is found in both eukaryotes and archaebacteria. |
| TC 9 | NA | DUF1818 | This presumed domain is found in a small family of cyanobacterial protein. These proteins are functionally uncharacterised. |
|  |  | DUF1997 | This family of proteins are functionally uncharacterised. |
|  |  | DUF2996 | This family of proteins has no known function. |
|  |  | DUF3155 | This family of proteins with unknown function appears to be restricted to Cyanobacteria. |
|  |  | DUF3611 | This family of proteins is found in bacteria and eukaryotes. Proteins in this family are typically between 180 and 205 amino acids in length. There are two completely conserved residues (W and G) that may be functionally important. |
|  | Photo | PSI_PsaF | Photosystem I (PSI) is an integral membrane protein complex that uses light energy to mediate electron transfer from plastocyanin to ferredoxin. Subunit III (or PSI-F) is one of at least 14 different subunits that compose the PSI complex. |
| TC 10 | AA | SelA | <NULL> |
|  | Carb | Gly_kinase | This is family of Glycerate kinases. |
|  | Lip | DAGK_cat | Diacylglycerol (DAG) is a second messenger that acts as a protein kinase C activator. The catalytic domain is assumed from the finding of bacterial homologues. YegS is the Escherichia coli protein in this family whose crystal structure reveals an active site in the inter-domain cleft formed by four conserved sequence motifs, revealing a novel metal-binding site. The residues of this site are conserved across the family. |
|  | NA | DUF227 | This family includes a large number of drosophila proteins of unknown function. The family also includes several C. elegans proteins. The alignment contains many histidines and aspartates that are conserved, suggesting a metal binding and possibly a phosphoesterase function (A Bateman pers. obs.). |
|  |  | DUF520 | Family of uncharacterised proteins. |
|  |  | DUF894 | This family consists of several bacterial proteins, many of which are annotated as putative transmembrane transport proteins. |
| TC 11 | AA | Na_Ala_symp | <NULL> |
|  | CoE | Glu_cys_ligase | Family of bacterial f glutamate-cysteine ligases (EC:6.3.2.2) that carry out the first step of the glutathione biosynthesis pathway. |
|  | E | NQR2_RnfD_RnfE | This family of bacterial proteins includes a sodium-translocating NADH-ubiquinone oxidoreductase (i.e. a respiration linked sodium pump). In Vibrio cholerae, it negatively regulates the expression of virulence factors through inhibiting (by an unknown mechanism) the transcription of the transcriptional activator ToxT. The family also includes proteins involved in nitrogen fixation, RnfD and RnfE. The similarity of these proteins to NADH-ubiquinone oxidoreductases was previously noted. |
|  |  | NQRA | This family consists of several bacterial Na(+)-translocating NADH-quinone reductase subunit A (NQRA) proteins. The Na(+)-translocating NADH: ubiquinone oxidoreductase (Na(+)-NQR) generates an electrochemical Na(+) potential driven by aerobic respiration. |
|  | NA | DUF2066 | This domain, found in various prokaryotic proteins, has no known function. |
|  |  | DUF484 | This family consists of several proteins of uncharacterised function. |
| TC 15 | NA | DUF3038 | This family of proteins with unknown function appear to be restricted to Cyanobacteria. |
|  |  | DUF3086 | This family of proteins with unknown function appears to be restricted to Cyanobacteria. |
|  |  | DUF3177 | Some members in this family of proteins are annotated as membrane proteins however this cannot be confirmed. Currently there is no known function. |
|  |  | DUF3571 | This family of proteins is functionally uncharacterised. This protein is found in bacteria and eukaryotes. Proteins in this family are typically between 85 to 97 amino acids in length. |
|  | Photo | Ycf4 | This family consists of hypothetical Ycf4 proteins from various chloroplast genomes. It has been suggested that Ycf4 is involved in the assembly and/or stability of the photosystem I complex in chloroplasts. |
| TC 29 | AA | OCD_Mu_crystall | This family contains the bacterial Ornithine cyclodeaminase enzyme EC:4.3.1.12, which catalyses the deamination of ornithine to proline. This family also contains mu-Crystallin the major component of the eye lens in several Australian marsupials, mRNA for this protein has also been found in human retina. |
|  |  | Pro_racemase | This family consists of proline racemase (EC 5.1.1.4) proteins which catalyse the interconversion of L- and D-proline in bacteria. This family also contains several similar eukaryotic proteins including Swiss:Q9NCP4 a sequence with B-cell mitogenic properties which has been characterised as a co-factor-independent proline racemase. |
|  | NA | DUF461 | Putative membrane or periplasmic protein. |
|  |  | DUF525 | Members of this family include the bacterial protein ApaG and the C termini of some F-box proteins (Pfam:PF00646). F-box proteins contain a carboxyl-terminal domain that interacts with protein substrates, so this family may be involved in protein-protein interaction. The function of ApaG proteins is unknown, but mutations in the Salmonella typhimurium ApaG homologue corD gives a phenotype of low-level cobalt resistance and decreased magnesium efflux by effects on the CorA magnesium transport system. |
| TC 46 | Carb | Rib_5-P_isom_A | This family consists of several ribose 5-phosphate isomerase A or phosphoriboisomerase A (EC:5.3.1.6) from bacteria, eukaryotes and archaea. |
|  | Def | Methylase_S | This domain is also known as the target recognition domain (TRD). Restriction-modification (R-M) systems protect a bacterial cell against invasion of foreign DNA by endonucleolytic cleavage of DNA that lacks a site specific modification. The host genome is protected from cleavage by methylation of specific nucleotides in the target sites. In type I systems, both restriction and modification activities are present in one heteromeric enzyme complex composed of one DNA specificity subunit (this family), two modification (M) subunits and two restriction (R) subunits. |
|  | NA | DUF192 | <NULL> |
