# Supplementary material for: Ecogenomic Perspectives on Domains of Unknown Function: Correlation-Based Exploration of Marine Metagenomes
Source: PLoS One. 2013 Mar 14;8(3):e50869. doi: 10.1371/journal.pone.0050869 (PMC3597751; doi:10.1371/journal.pone.0050869)
Supplement: Table S10 — DUFs with updated statuses in Pfam v26 and their location in transitivity clusters (TCs) from both the unstandardized (UM; Figure 3 ) and standardized (SM; Figure 4 ) datasets. (DOC) [file pone.0050869.s011.doc]

Table S10: DUFs with updated statuses in Pfam v26 and their location in transitivity clusters (TCs) from both the unstandardized (UM; Figure 3) and standardized (SM; Figure 4) datasets.

| **Pfam v24 ID** | **Status in Pfam v26** | **UM TC** | **SM TC** |
| --- | --- | --- | --- |
| DUF989 | Haem-binding domain towards C-terminus merged with Cytochrome_C family. | TC3 | -- |
| DUF894 | Renamed to MFS_3, a transmembrane secretion effector domain family. The ferric chelator, enterobactin, and an antibiotic resistance protein are transported by MFS_3. | TC10 | -- |
| DUF88 | Renamed to NYN, found in bacterial YacP-like proteins. Predicted RNAses. | No associations | -- |
| DUF785 | Renamed to Zn_protease, a putative ATP-dependent zinc protease family | No associations | -- |
| DUF74 | Renamed to YbjQ_1, a putative heavy-metal-binding domain family | No associations | -- |
| DUF583 | Renamed to Bactofilin, a family of polymer-forming cytoskeletal proteins conserved in bacteria. | TC110 | -- |
| DUF55 | Renamed to EVE, a ASCH/PUA-related domain with putative RNA-binding activity. | TC1 | -- |
| DUF548 | Renamed to SAM_MT, a family of SAM-dependent methyltransferases | TC49 | -- |
| DUF407 | Renamed to CP_ATPgrasp_1, an ATP-grasp family with suggested function in a peptide synthesis and modification system. | TC30 | TC9 |
| DUF404 | Merged with CP_ATPgrasp_1, formerly DUF407. | TC30 | TC9 |
| DUF403 | Renamed to Alpha-E, a predicted alpha-helical domain with suggested function in a peptide synthesis and modification system. | TC30 | TC9 |
| DUF37 | Renamed to Haemolytic, a domain with haemolytic activity. | TC7 | No associations |
| DUF299 | Renamed to Kinase-PPPase, a family of regulatory proteins with kinase and phosphorylase activity. | TC1 | -- |
| DUF28 | Renamed to Transcrip_reg, a family which negatively regulates a quorum-sensing response regulator. | TC1 | -- |
| DUF227 | Renamed to EcKinase, an insect ecdysteroid kinase. | TC10 | -- |
| DUF2159 | Merged into LptE, part of a transport complex involved in lipopolysaccharide assembly on the outer membrane of Gram negative bacteria. | No associations | -- |
| DUF205 | Renamed to G3P_acyltransf, a family of glycerol-3-phosphate acyltransferases. | TC1 | -- |
| DUF2010 | Renamed to PGP_phosphatase, a family of proteins involved in cardiolipin biosynthesis. | No associations | TC14 |
| DUF1994 | Dead Pfam family | -- | -- |
| DUF185 | Renamed to Methyltransf_28, a putative SAM-dependent methyltransferase. | TC1 | -- |
| DUF1790 | Merged into YbjN, a putative bacterial sensory transduction regulator family. | No associations | -- |
| DUF151 | Renamed to DNase-RNase, a bifunctional nuclease family. | No associations | -- |
| DUF149 | Renamed to YbaB_DNA_bd, a family of DNA-binding domains which may be involved in replicative DNA repair. | TC1 | -- |
| DUF143 | Renamed to Oligomerisation, a family of domains needed for the oligomerisation of ATP synthase subunit 9 in yeast. | TC1 | -- |
| DUF140 | Renamed to Permease, a family of domains with permease functionality. | No associations | -- |
| DUF1239 | Renamed to LptC, part of a transport complex involved in lipopolysaccharide assembly on the outer membrane of Gram negative bacteria. | TC1 | -- |
| DUF1185 | Renamed to AA_synth, a domain family with a suggested role in amino acid synthesis. | TC122 | -- |
| DUF1022 | Renamed Mito_fiss_Elm1, involved in mitochondrial fission in plants. Unknown function in bacteria. | TC3 |  |
| DUF1008 | Renamed ChuX_HutX, linked to haem utilisation | TC78 | No associations |
