# Supplementary figures and images for: Ecogenomic Perspectives on Domains of Unknown Function: Correlation-Based Exploration of Marine Metagenomes
Source: PLoS One. 2013 Mar 14;8(3):e50869. doi: 10.1371/journal.pone.0050869 (PMC3597751; doi:10.1371/journal.pone.0050869)

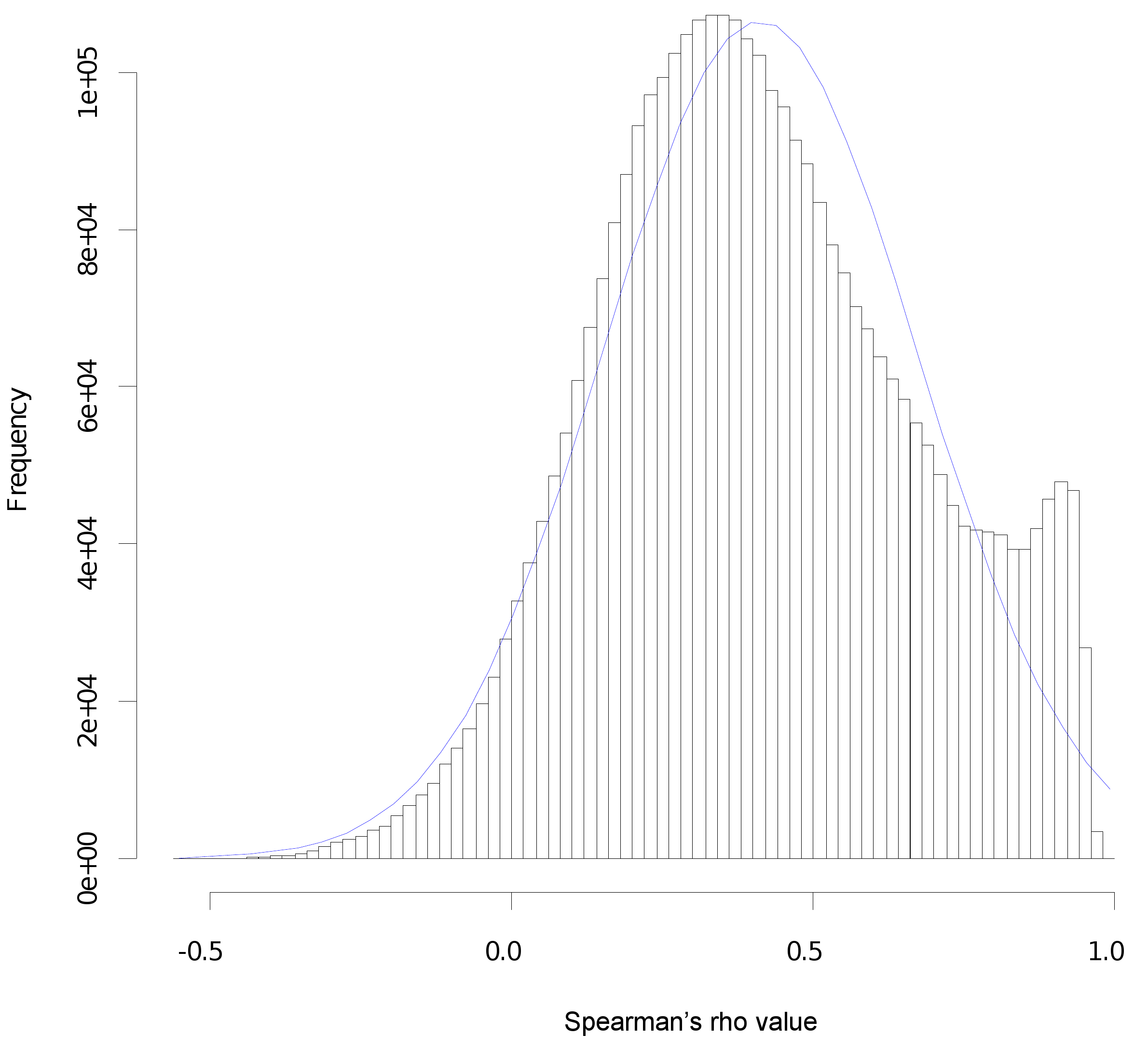

Supplement: Figure S1 — Histogram of correlation strength between unstandardized abundances of Pfam domains across GOS metagenomes. The distribution's mean and standard deviation were ∼0.41 and ∼0.26 respectively. A normal distribution with equal mean and standard deviation is indicated by a blue contour. (TIF) [file pone.0050869.s012.tif]

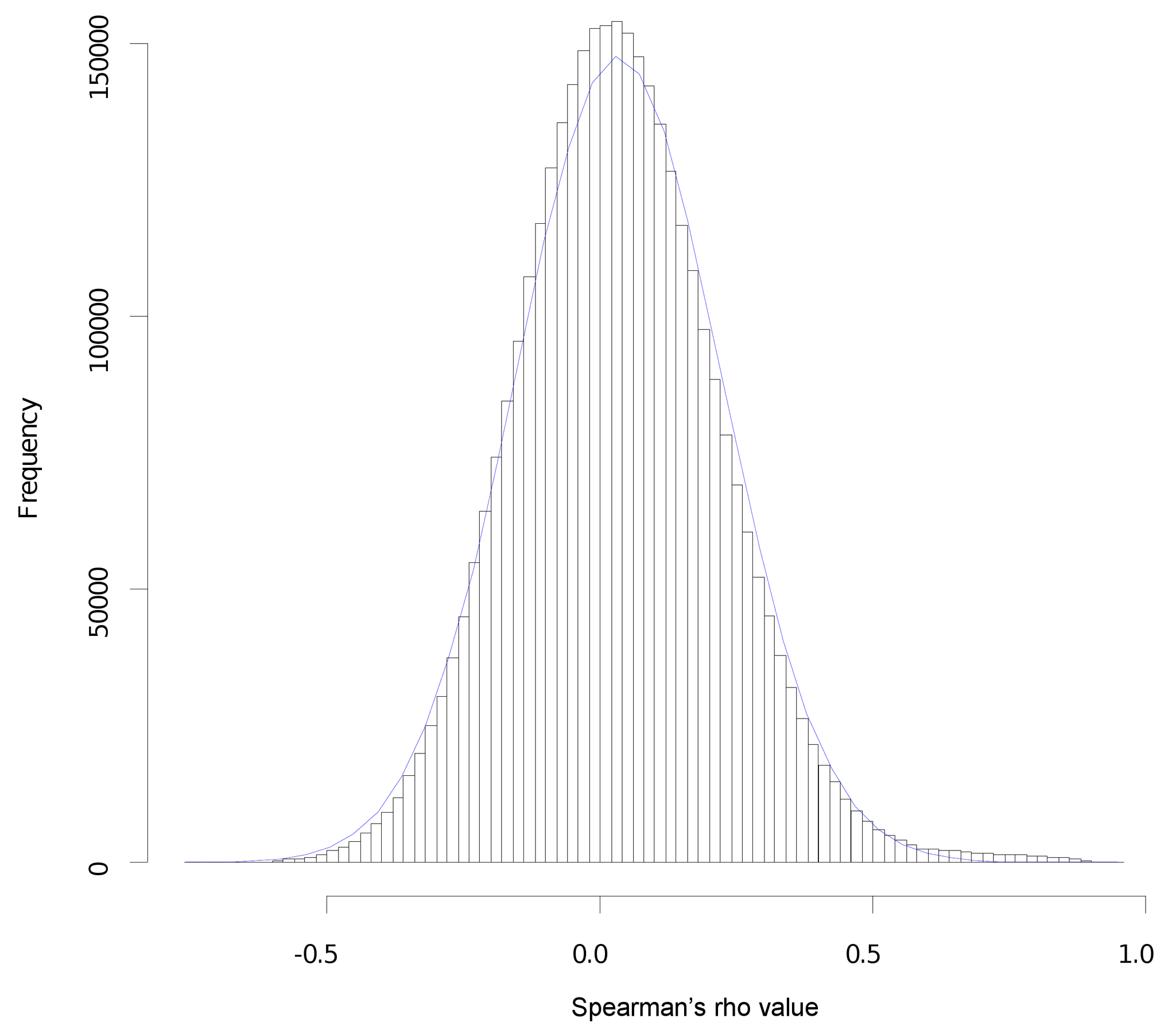

Supplement: Figure S2 — Histogram of correlation strength between abundances of Pfam domains across GOS metagenomes, standardized by site maxima. The distribution's mean and standard deviation were ∼0.03 and ∼0.19 respectively. A normal distribution with equal mean and standard deviation is indicated by a blue contour. (TIF) [file pone.0050869.s013.tif]

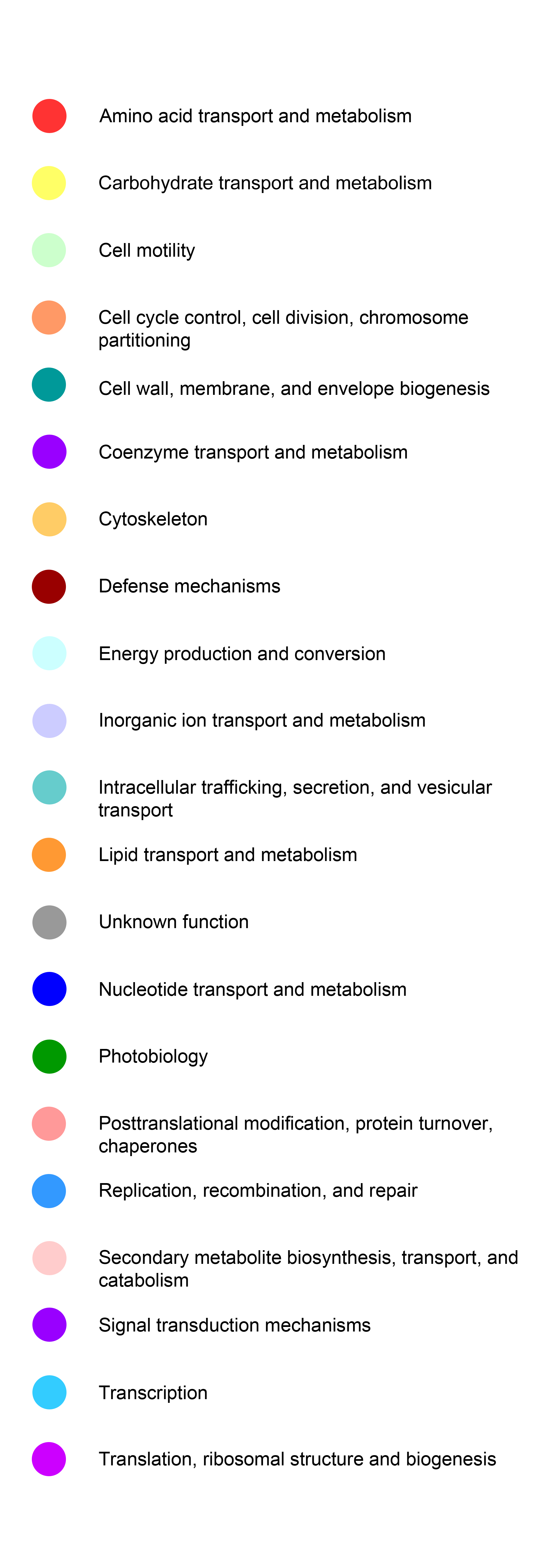

Supplement: Figure S7 — Color key for network nodes. (TIF) [file pone.0050869.s018.tif]
